# AutoComBat: a generic method for harmonizing MRI-based radiomic features

**DOI:** 10.1038/s41598-022-16609-1

**Published:** 2022-07-26

**Authors:** Alexandre Carré, Enzo Battistella, Stephane Niyoteka, Roger Sun, Eric Deutsch, Charlotte Robert

**Affiliations:** 1 Université Paris-Saclay, Institut Gustave Roussy, Inserm, Radiothérapie Moléculaire et Innovation Thérapeutique, 94800 Villejuif, France; 2 Institut Gustave Roussy, Département de Radiothérapie, 94800 Villejuif, France; 3 Université Paris-Saclay, CentraleSupélec, Mathématiques et Informatique pour la Complexité et les Systèmes, 91190 Gif-sur-Yvette, France; 4 Université Paris-Saclay, CentraleSupélec, Inria, Gif-sur-Yvette, France

**Keywords:** Cancer imaging, Computational science, Tumour biomarkers

## Abstract

The use of multicentric data is becoming essential for developing generalizable radiomic signatures. In particular, Magnetic Resonance Imaging (MRI) data used in brain oncology are often heterogeneous in terms of scanners and acquisitions, which significantly impact quantitative radiomic features. Various methods have been proposed to decrease dependency, including methods acting directly on MR images, i.e., based on the application of several preprocessing steps before feature extraction or the ComBat method, which harmonizes radiomic features themselves. The ComBat method used for radiomics may be misleading and presents some limitations, such as the need to know the labels associated with the “batch effect”. In addition, a statistically representative sample is required and the applicability of a signature whose batch label is not present in the train set is not possible. This work aimed to compare a priori and a posteriori radiomic harmonization methods and propose a code adaptation to be machine learning compatible. Furthermore, we have developed AutoComBat, which aims to automatically determine the batch labels, using either MRI metadata or quality metrics as inputs of the proposed constrained clustering. A heterogeneous dataset consisting of high and low-grade gliomas coming from eight different centers was considered. The different methods were compared based on their ability to decrease relative standard deviation of radiomic features extracted from white matter and on their performance on a classification task using different machine learning models. ComBat and AutoComBat using image-derived quality metrics as inputs for batch assignment and preprocessing methods presented promising results on white matter harmonization, but with no clear consensus for all MR images. Preprocessing showed the best results on the T1w-gd images for the grading task. For T2w-flair, AutoComBat, using either metadata plus quality metrics or metadata alone as inputs, performs better than the conventional ComBat, highlighting its potential for data harmonization. Our results are MRI weighting, feature class and task dependent and require further investigations on other datasets.

## Introduction

Either for clinical diagnosis, prognosis, and therapy assessment of brain pathologies or neuroscience research, magnetic resonance (MR) imaging is of prime importance. However, MR images are subject to wide quantitative variations inherent to this imaging modality, i.e. MR data acquired for the same patient but on different sites or scanners yield to different MR images^1–3^. Additional differences can also be attributed to artifacts such as bias field inhomogeneities, noise, motion, ghosting, or spike^4–8^. The major limitation of MRI compared to other imaging modalities is that the signal intensity described in grey values is arbitrary, unlike computerized tomography (CT) and positron emission tomography (PET), which are described by quantitative scales such as Hounsfield units (HU) or semi-quantitative scales such as standardized uptake value (SUV).

Radiomics is a rapidly evolving field based on computer vision techniques. It refers to the high-throughput extraction of numerous quantitative features (including shape, intensity, or texture) from images that can be used as potential biomarkers^9–11^. It has shown promise in brain cancer detection, diagnosis, molecular mutation characterization, prognosis, and outcome prediction in oncology^12–18^. However, radiomic features are well recognized to be vulnerable to differences in MR imaging^19–22^. This weakness is hampering the integration of data from different centers in predictive analysis and/or machine learning (ML) algorithms and the construction of subsequent robust models. To voluntary ignore scanner-induced data heterogeneity, most neuroimaging studies have traditionally been limited to datasets from a single center^23^. In recent years, there has been an increasing trend towards the collection and sharing of neuroimaging data through the establishment of multi-institutional databases^24,25^. This effort to collect data covering a wide range of machine types and broad spectrum population (demographic) is essential for the development of diagnostic and prognostic biomarkers to enable robust translation of research into clinical practice. As a consequence, there is a strong and pressing need for standardization and/or harmonization^26,27^. Compensation for these effects can be seen at three different levels: (i) Image acquisition, (ii), Image processing and, (iii) Feature adjustment.

One of the solutions is to consider standardized procedures regarding imaging protocols to reduce the institutional effect and obtain more constant images^28^. However, this solution seems difficult to envisage on a large scale, since it requires convincing a large majority of centers to adopt the same protocol, which can be long and complicated to enforce. Moreover, the issue would remain for retrospective studies and would not negate the manufacturer/device effect either. The second solution is to consider a well-defined image processing pipeline that can harmonize images post-acquisition. A classical image processing process includes at least a bias field correction, an isotropic voxel resampling, a skull stripping and finally a standardization of the brain image intensities, which can be performed by the Nyùl et al., Hybrid White Stripe or Z-Score methods^29^. This approach is suitable for deep learning segmentation approaches to feed the network and shows promising results in radiomic studies^29,30^. Recently, deep learning techniques using fully convolutional neural networks for contrast harmonization have emerged, but may require an overlap cohort of patients scanned with the respective protocols^31^. Besides, this may require patients to be reimaged, which may be impractical or impossible, or may limit the training data. Finally, the third solution consists in applying a correction directly to the derived radiomic features without any pre-correction on the image. The breakthrough approach in this category is ComBat, a batch effect correction tool originally used in genomics^32^ and first adapted to harmonize diffusion MRI^33^. Recent studies applied to MRI showed that this method would lead to an efficient reduction of discrepancies in values of radiomic features between centers and improve the accuracy of experiments with data from multiple scanners^34,35^. This method has, however, several drawbacks as the need for a representative statistical sample to estimate the batch effect parameters. Furthermore, this method does not meet two essential criteria for a machine learning applicability. First, the correct application of a feature scaling method requires applying the estimators learned on the training set to the test set. Second, if we want to see the applicability of a model and its translation into clinical practice, it has to be generalizable and make predictions on a single image from a site or scanner that was not part of the training set. In addition, it may not always be simple to define the notion of “batch effect”, since this effect can be seen at different levels: (i) site/center, (ii) scanner, (iii) variation in scanner parameters. Ideally, the determination of batch effect labels should correspond to the grouping of imaging data with similar image qualities, often associated with similar acquisition and reconstruction parameters.

We thus developed a method that should allow the applicability of feature adjustments in a highly multi-centric radiomic machine learning context. This method called AutoComBat allows a sample to be assigned to a specific batch by a constrained clustering method. In this method, the batch label can be defined by metadata summarizing the scanner and the associated acquisition characteristics (DICOM tags) or by image quality metrics measurements.

In addition to providing a proof of concept for the applicability of the AutoComBat method, we aimed to answer the question whether the harmonization strength of a classical preprocessing method is comparable to the ones of the ComBat and AutoComBat methods in decreasing relative standard deviation of radiomic features extracted from white matter. Second, we studied their respective performances on a classification task in machine learning.

## Material and methods

### Dataset

Preoperative scans previously extracted from the The Cancer Imaging Archive (TCIA)^36^ including both glioblastoma (TCGA-GBM, n = 135) and low-grade-glioma (TCGA-LGG, n=108) were considered^24,37,38^. All methods were performed in accordance with the relevant guidelines and regulations (Declaration of Helsinki). Selected DICOM files were pre- and post-contrast T1-weighted (T1w and T1w-gd), T2-weighted (T2w), and T2 Fluid-Attenuated Inversion Recovery (FLAIR) volumes (T2w-flair). These data presented high heterogeneity as they were collected from 11 different centers. Data from the three centers showing the lowest sample numbers were removed to ensure at least 5 samples in the training set after stratified data splitting^39,40^ (see section Batch effect adjustment method and subsection Empirical Bayes method). The other criteria was the availability of sex and age information, which was not the case for one patient. At the end, 232 samples were kept corresponding to 125 GBM and 107 LGG from 8 different centers. Table 1 summarizes centers and associated numbers of patients included in our study.

**Table 1:** Institutional information of patients of Bakas et al.^24^ and patients selected in our study.

Collection	Institutions	N	N selected	TCGA-ID
TCGA-GBM	Henry Ford Hospital, Detroit, MI	46	46	TCGA-06
	CWRU School of Medicine, Cleveland, OH	9	9	TCGA-19
	University of California, San Francisco, CA	22	22	TCGA-08
	Emory University, Atlanta, GA	6	0	TCGA-14
	MD Anderson Cancer Center, Houston, TX	25	25	TCGA-02
	Duke University School of Medicine, Durham, NC	10	9	TCGA-12
	Thomas Jefferson University, Philadelphia, PA	14	14	TCGA-76
	Fondazione IRCCSInstituto Neuroligico C. Besta, Milan, Italy	3	0	TCGA-27
TCGA-LGG	St Joseph Hospital/Medical Center, Phoenix, AZ	29	29	TCGA-HT
	Henry Ford Hospital, Detroit, MI	52	52	TCGA-DU
	Case Western Reserve University, Cleveland, OH	10	10	TCGA-FG
	Thomas Jefferson University, Philadelphia, PA	16	16	TCGA-CS
	University of North Carolina, Chapel Hill, NC	1	0	TCGA-EZ
Total		243	232	

* TCGA* The Tumor Genome Atlas.

### Batch effect adjustment method

The ComBat harmonization method was originally designed for the field of genetics to overcome the “batch effect” observed in microarray analysis^32^. The term “batch effect” refers to non-biological noise which affects samples to be analyzed. It can be due to diverse factors such as operator’s methodology, sequencing technology, time of day of measurements, etc., and makes difficult direct comparisons. In radiomic studies, the different “batches” can be related to different imaging protocols or devices. One of the advantages is that ComBat can harmonize radiomic features by considering the batch as a covariate, while preserving the variance due to other known covariates such as gender or age for example.

#### Model-based location/scale adjustments

ComBat harmonisation is derived from the location (mean) and scale (variance) (L/S) method, in which the main idea is to transform the data of each batch so that they end up with the same mean and/or variance and thus eliminate the error introduced by the differences between the batches. For example, let $Y_{i j f}$ represents the value corresponding to feature *f* for sample *j* from batch *i*. The L/S adjustment method models the feature’s value as:

1 $$\begin{aligned} Y_{i j f}=\alpha _{f}+X \beta _{f}+\gamma _{i f}+\delta _{i f} \varepsilon _{i j f}, \end{aligned}$$

where $\alpha _{f}$ is the overall feature value, *X* is a matrix for the covariates of interest, and $\beta _{f}$ is the vector of regression coefficients corresponding to *X*. The error terms, $\varepsilon _{i j f}$, can be assumed to follow a Normal distribution with expected values of mean zero and variance $\sigma _f^2$. The $\gamma _{i f}$ and $\delta _{i f}$ respectively represent the additive and multiplicative batch effects corresponding to batch i for feature f. The estimation of these two terms allows to determine the value adjusted for the batch effect using the following equation:

2 $$\begin{aligned} Y_{i j f}^{*}=\frac{Y_{i j f}-\widehat{\alpha }_{f}-X \widehat{\beta }_{f}-\widehat{\gamma }_{i f}}{\widehat{\delta }_{i f}}+\widehat{\alpha }_{f}+X \widehat{\beta }_{f}, \end{aligned}$$

where $\widehat{\alpha }_{f}$, $\widehat{\beta }_{f}$, $\widehat{\gamma }_{i f}$ and ${\widehat{\delta }_{i f}}$ are estimators of the parameters ${\alpha }_{f}$, ${\beta }_{f}$, ${\gamma }_{i f}$ and ${{\delta }_{i f}}$.

#### Empirical Bayes method

ComBat method uses an empirical Bayes (EB) framework to better adjust the parameter estimates $\widehat{\gamma }_{i f}$ and ${\widehat{\delta }_{i f}}$ in case of limited sample sizes, making the hypothesis that batch effect affects features in similar ways. The minimum number of samples in each batch has been defined as 5^39,40^. There exist both a parametric and a non-parametric approaches. We give here a concise explanation about the parametric one, and additional details can be found in the original publication^32^. The first step in EB is to standardize the data by features to ensure they have a similar overall mean and variance. The standardized feature value $Z_{i j f}$ is given by:

3 $$\begin{aligned} Z_{i j f}=\frac{Y_{i j f}-\widehat{\alpha }_{f}-\mathrm {X} \widehat{\beta }_{f}}{\widehat{\sigma }_{f}} \end{aligned}$$

where $Y_{i j f}$, $\widehat{\alpha }_{f}$ and $\widehat{\sigma }_{f}$ are respectively the raw feature value, feature-wise mean and standard deviation estimates. $X\widehat{\beta }_{f}$ denotes the model’s possible non-batch related covariates and coefficients. The standardized feature value $Z_{i j f}$ is assumed to be normally distributed according to $Z_{i j f} \sim N\left( \gamma _{i f}, \delta _{i f}^{2}\right)$, where the batch effect parameters are assumed with the following prior distributions $\gamma _{i f} \sim N\left( Y_{i}, \tau _{i}^{2}\right)$ and $\delta _{i f}^{2} \sim$ Inverse Gamma $\left( \lambda _{i}, \theta _{i}\right)$. The moments method is used to estimate the hyperparameters $\gamma _{i}, \tau _{i}^{2}, \lambda _{i}, \theta _{i}$ empirically from standardized data. The EB estimates for the batch effect parameters, $\gamma _{i f}^{*}$ and $\delta _{i f}^{2 *}$, can be derived by the conditional posterior means given the distributional assumptions mentioned previously. Henceforth, the EB batch effect adjusted features $\gamma _{i j f}^{*}$ can be calculated in a similar way to Eq. (2) as follows:

4 $$\begin{aligned} \gamma _{i j f}^{*}=\frac{\widehat{\sigma }_{f}}{\widehat{\delta }_{i f}^{*}}\left( Z_{i j f}-\widehat{\gamma }_{i f}^{*}\right) +\widehat{\alpha }_{f}+X \widehat{\beta }_{f} \end{aligned}$$

The ComBat method, as described in the original paper, centers the data on the overall, grand mean and pooled variance of all samples. This results in a harmonized location-shifted data matrix that no longer corresponds to any initial batch which can lead to a loss of physical meaning. A modified version proposed that a reference batch label can be chosen to shift each sample to the mean and variance of this reference^41^. This is accomplished by simply changing the estimates of the standardization mean and variance, $\widehat{\alpha }_{f}$ and $\widehat{\sigma }_{f}$ [Eq. (3)], to batch estimates, $\widehat{\alpha }_{i f}$ and $\widehat{\sigma }_{i f}$. Thus, as part of the development of a machine learning model, ComBat’s model parameters (e.g., $\widehat{\alpha }_{f}$, $\widehat{\sigma }_{f}$, $\widehat{\beta }_{f}$, $\gamma _{i f}^{*}$ and $\delta _{i f}^{2 *}$ for the conventional ComBat or $\widehat{\alpha }_{if}$, $\widehat{\sigma }_{if}$, $\widehat{\beta }_{f}$, $\gamma _{i f}^{*}$ and $\delta _{i f}^{2 *}$ for the modified version), learned from a training set, should not involve any test set data, but should be stored for later transfer to unseen data.

### AutoComBat approach

We propose in this section, AutoComBat, based on the hypothesis that batch labels can be deduced from image metadata (DICOM tags) and/or image quality metrics.

#### DICOM tags and image quality metrics extraction

In the present work, two main classes of information were extracted from the DICOM files. Table 2 summarizes the DICOM tags of interest and the image quality metrics deduced from the data matrices themselves with their mathematical formulation.

- Metadata: Information extracted from the header of the DICOM file describing the MR device and acquisition parameters (i.e. Magnetic field, manufacturer, voxel sizes, ...). In total, 15 tags were considered (Table 2).
- Quality metrics: These metrics have recently been proposed to quantify the batch scanner effect in MRI as well as to detect artifacts^42^. This class includes statistical measures (e.g., range, variance, coefficient of variation) as well as second-order statistics and filter-based measures (e.g., contrast per pixel (CPP), entropic focus criterion (EFC), signal-to-noise ratios corresponding to different regions). In total, 15 quality metrics were considered (Table 2).

**Table 2:** Summary table of metadata and quality metrics extracted from the raw DICOM files.

Type	Name	Description	Tags
Metadata	Rows	Number of rows in the image	0028,0010
	Columns	Number of columns in the image	0028,0011
	Vox_X	Voxel resolution in x plane	0028,0030
	Vox_Y	Voxel resolution in y plane	0028,0030
	Vox_Z	Voxel resolution in z plane	0018,0050
	PixelBandwidth	Reciprocal of the total sampling period, in hertz per pixel	0018,0095
	Manufacturer	Manufacturer of the equipment	0008,0070
	ModelName	Model name of the manufacturer of the equipment	0008,1090
	MagneticField	Nominal field strength of the MR magnet, in Tesla	0018,0087
	EchoNumbers	Echo number used to generate the image	0018,0086
	EchoTime	Time in ms between the middle of the excitation pulse and the peak of the echo produced (kx=0)	0018,0081
	EchoTrainLength	Number of lines in k-space acquired per excitation per image	0018,0091
	InversionTime	Time in ms between the middle of the inverting RF pulse and the middle of the excitation pulse to detect the amount of longitudinal magnetization	0018,0082
	RepetitionTime	The period of time in ms between the beginning of a pulse sequence and the beginning of the succeeding (essentially identical) pulse sequence	0018,0080
	FlipAngle	Steady state angle in degrees by which the magnetic vector is flipped with respect to the magnetic vector of the primary field	0018,1314

Type	Name	Description	Formula
Quality metrics	Mean	Mean of the foreground	$\dfrac{F}{n}$
	Range	Range of the foreground	$\max (F)-\min (F)$
	Variance	Variance of the foreground	$\sigma _{F}^{2}$
	PCV	Percent coefficient of variation: coefficient of variation of the foreground for shadowing and inhomogeneity artifacts^49^	$\dfrac{\sigma _{F}}{\mu _{F}}$
	CPP	Contrast per pixel: mean of the foreground filtered by a 3x3 2D Laplacian kernel for shadowing artifacts^50^	${\text {mean}}({\text {conv2}} (\mathrm {F}, \mathrm {f}_{1}))$, $$\mathrm {f}_{1} = \begin{bmatrix} - 1 &{} -1 &{} -1 \\ -1 &{} 8 &{} -1 \\ -1 &{} -1 &{} -1 \end{bmatrix}$$
	PSNR	Peak signal to noise ratio of the foreground^51^	$10 \log \dfrac{\max ^{2}(F)}{{\text {MSE}}(F, f_{2})}$, $f_{2}$ is a 5 × 5 × 5 median filter
	SNR1	Foreground standard deviation (SD) divided by background SD^52^	$\dfrac{\sigma _{F}}{\sigma _{B}}$
	SNR2	Mean of the foreground patch divided by background SD^53^	$\dfrac{\mu _{F_{P}}}{\sigma _{B}}$
	SNR3	Foreground patch SD divided by the centered foreground patch SD	$\dfrac{\mu _{F_{P}}}{\sigma _{F_{P}}\mu _{F_{P}}}$
	SNR4	Mean of the foreground patch divided by mean of the background patch	$\dfrac{\mu _{F_{P}}}{\sigma _{B_{P}}}$
	CNR	Contrast to noise ratio for shadowing and noise artifacts: mean of the foreground and background patches difference divided by background patch SD^52^	$\dfrac{\mu _{F_{P}}-B_{P}}{\sigma _{B_{P}}}$
	CVP	Coefficient of variation of the foreground patch for shading artifacts: foreground patch SD divided by foreground patch mean	$\dfrac{\sigma _{F_{P}}}{\mu _{F_{P}}}$
	CJV	Coefficient of joint variation between the foreground and background for aliasing and inhomogeneity artifacts^54^	$\dfrac{\sigma _{F}+\sigma _{B}}{|\mu _{F}-\mu _{B}|}$
	EFC	Entropy Focus criterion for motion artifacts^53^	$- \sum _{i=1}^n \dfrac{F_i}{F_\text {max}} \ln [\dfrac{F_i}{F_\text {max}}]$, $F_{\mathrm {max}}=\sqrt{\sum _{i, j} F^{2}(i, j)}$
	FBER	Foreground-background energy ratio for ringing artifacts^55^	$\dfrac{{\text {median}}(|F|^{2})}{{\text {median}}(|B|^{2})}$

F is Foreground intensity voxels ($F=\sum ^{n}_{i=1}\dfrac{v_{f_{i}}}{n}$) with $v_{f_{i}}, i^{th}$ foreground voxels. B is Background intensity voxels ($B=\sum ^{n}_{i=1}\dfrac{v_{b_{i}}}{n}$) with $v_{b_{i}}, i^{th}$ background voxels.

$F_{P}$ is Foreground random patch voxels (n = 5000, with a 5 × 5 × 5 patch-size). $B_{P}$ is Background random patch voxels (n = 5000, with a 5 × 5 × 5 patch-size).

#### Determination of batch effect labels using clustering

Based on the extracted information, AutoComBat uses K-Means clustering with constraints^43^ on the minimum cluster size to ensure the condition that ComBat uses a statistically representative sample from each identified batch. We set the minimum cluster size to 5 samples in this work as demonstrated to be statistically representative in ComBat^39,40^, but this value can be changed in our approach. The features used to determine the batch effect were processed in two different ways, depending on whether they were discrete (Manufacturer, model name) or continuous (Voxel sizes, echo time, ...). The discrete variables were one-hot encoded, and a NaN category was added to account for the case where no missing value was encountered during training but could be experienced during the prediction phase. The continuous variables were treated by subtracting the mean and scaling to unit variance. The K-Means constrained clustering was able to take into account missing values. For this, the missing values were initialized to the mean of their column, and an expectation-maximization (EM) algorithm was executed until convergence of stability in the label prediction. We set the threshold for the missing features to 25%, which means that for a given feature, 75% of the data must be present for training. Furthermore, we added the possibility to embed a feature reduction before clustering, either with Principal Component Analysis (PCA)^44^ or Uniform Manifold Approximation and Projection (UMAP)^45^. To determine the optimal number of clusters, the elbow method of the Yellowbrick library was used^46^. The elbow method runs the K-Means constrained clustering on the dataset for all possible values of K. Then, for each value of K, a metric is computed to evaluate quality of the clusters. By default, the scoring metric is the distortion, which calculates the sum of the squared distances from each point to its assigned cluster center. However, two other metrics can be used: the Silhouette score and the Calinski-Harabasz score. The Silhouette score calculates mean ratio of intra-cluster and nearest-cluster distance, while the Calinski Harabasz score calculates the ratio of dispersion between and within the clusters. The optimal value of K was determined automatically using the ”knee point detection algorithm” which allows to determine the elbow, i.e. the point of inflection^47^. To use a reference batch in ComBat, our approach estimated the most relevant cluster for this role as the one with the lowest within-cluster sum-of-squares (WCSS), defined as the sum of the squared distances between each member of the cluster and its centroid.

We implemented ComBat and AutoComBat in Python compatible with scikit-learn^48^ to facilitate subsequent machine learning projects. ComBat can use EB or more simpler L/S method. When EB is chosen, adjustments can be done in a parametric or non-parametric way. A reference batch can also be set in case the user prefers to use the modified version of ComBat. AutoComBat benefits from the ComBat inheritance. The code is available at the following address: https://github.com/Alxaline/ComScan.

To extract the image quality metrics and the metadata from the DICOM files, we have also developed a Python package available at the following address: https://github.com/Alxaline/QAnT, mainly based on the image quality metric available in MRQy^42^ (https://github.com/ccipd/MRQy). The main difference is that we extract the metrics directly per 3D patch and not by an average on 2D slices. Moreover, the metadata extraction is fully customizable, and the code has been accelerated by multiprocessing.

### Image processing approach

Image preprocessing is an alternative approach to reduce the batch effect by applying various correction steps prior to the extraction of the radiomic features. The pipeline that we used on the DICOM files included 4 steps: bias field correction, coregistration (voxel size resampling), skull-stripping and z-score normalization^29^. First, the N4 bias field correction was applied to all MRI images considering the head area as the region of interest^4^. Then, for each patient, the T1w image was registered to the T1w SRI-24 atlas reoriented to the LPS (left-posterior-superior) coordinate system^56^ using an affine transformation and a B-Spline interpolation. The resulting image, T1w$_{reg}$, had a 1x1x1 $mm^3$ voxel size. The other MR images, i.e. T1w-gd, T2w and T2w-flair, were co-registered to T1w$_{reg}$. The modalities were then skull-stripped to keep only the brain^57^. Finally, the z-score normalization was applied to the brain voxels by setting the mean to zero and the variance to one.

The package used for preprocessing is the cBrainMRIPrePro Python package available at the following address: https://github.com/Alxaline/cBrainMRIPrePro. This in-house package uses ANTsPy^58^ and HD-bet^57^ and enables the preprocessing of anatomical MR images in the form of a straightforward pipeline.

### Radiomic feature extraction

The extraction of radiomic features was performed using the Python library Pyradiomics^59^ v3.0.1. A total of 91 features were extracted including 18 first-order and 73 second-order features compliant with the Image Biomarker Standardization Initiative (IBSI), except for the first-order feature Kurtosis, where Kurtosis is calculated using -3 and +3 in the IBSI and PyRadiomics standards respectively^26^. The second-order features corresponded to 22 features from the Grey Level Co-occurrence Matrix (GLCM), 16 features from the Grey Level Run Length Matrix (GLRLM), 16 features from the Grey Level Size Zone Matrix (GLSZM), 5 features from the Neighborhood Grey Tone Difference Matrix (NGTDM) and 14 features from the Grey Level Dependence Matrix (GLDM). Prior to feature extraction, an intensity shifting of 300 was performed to guarantee that the majority of voxel intensities were positive. For each combination, i.e. MR image plus region of interest (white matter patches or whole tumor, see section Experiments and analysis), extraction was performed according to a specific bin width. The intensity ranges from the whole patient dataset were used to calculate the optimal bin width leading to 32 bins, which was a reasonable balance^29^.

### Experiments and analysis

The experiments first sought to assess the strength of harmonization of each method on the radiomic features. Next, we evaluated the impact of these three methods on a problem of classifying brain tumors into two different categories: GBM and LGG.

**Figure 1:**
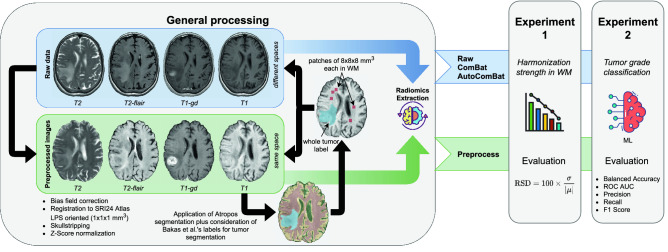
Study design.

For the two experiments, we separated the data into three sets: Training, Validation, and Testing. This strategy allowed us to avoid overly optimistic results due to overfitting. Also, this strategy was prefered to k-fold cross validation to meet the requirements of the ComBat method, whose philosophy is to have at least five samples per batch (here, considered as the center) in the training set and due to the fact that the test cannot contain a sample of a batch label that has not been seen in the training phase. Also, a leave-one-out cross-validation strategy was not considered due to the computational cost. Thus, our validation set was used to maximize the optimization metric, and the test set was used to report the final performance of the model. Like any normalization step in machine learning, ComBat and AutoComBat were applied after splitting the data. The split was stratified by tumor type and the repartition was as follows: 130, 44, and 58 samples for training, validation, and testing, respectively. The design of the study is illustrated in Fig. 1.

#### Experiment 1: harmonization strength

White matter areas are distinguished by vast homogeneous regions with only minor variations in intensity between patients^60^. We exploited this consideration to hypothesize that the variation of radiomic feature values extracted from this area should be minimal between patients when the machine effect is reduced. To that end, a label map was created for every patient using Atropos which is a finite mixture modeling (FMM) segmentation approach^61^. Atropos made possible to extract three brain regions: the cerebral spinal fluid (CSF), the grey matter (GM) and the white matter (WM) automatically. The mask that defined the area to be labeled corresponded to the brain mask subtracted by the total tumor mask. The whole tumor corresponded to the union of the enhancing tumor (ET), necrotic tumor (NEC), non enhancing tumor (NET) and peritumoral edema (ED), as defined by Bakas et al.^24^. The Atropos label maps were all manually verified by an image scientist (A.C). Thirty randomly located 8x8x8 mm$^{3}$ patches were considered in the segmented white matter region as regions of interest (ROI). All ROI, i.e. the whole tumor and the white matter patches, were also remapped in each space of each raw MR image. To consider only the batch effect and preserve biological associations in ComBat and AutoComBat, gender, age, and tumor type were kept as covariables. Age was treated as a categorical variable and two categories were considered: above and below 50 years of age, since they have previously been shown to generate differences in white matter MR signal^62^. This was necessary to meet the minimum sample size of 5 per category. For ComBat and AutoCombat, optimization was performed for each MR image type with a grid search to sift through each combination of hyperparameters. The number of combinations evaluated was 27 and 54 for ComBat and AutoComBat, respectively. The parameter space used for the grid search is given in Table S1. The strength of the correction was assessed by the minimization of the objective function described in Eq. (5) which corresponds to the average of the relative standard deviation (RSD) over the whole set of radiomics features.

5 $$\begin{aligned} \mathscr {L}=\frac{1}{n}\sum _{f=1}^nRSD_f=\frac{1}{n}\sum _{f=1}^n\frac{\sigma _{f}}{\mid \mu _{f} \mid } \times 100 \end{aligned}$$

where *n* is the total number of radiomic features, $\sigma _{f}$ is the standard deviation and $\mu _{f}$ is the mean of feature *f*. The 95% confidence interval (CI) of the RSD was computed for each feature using bootstrapping with 1000 rounds.

#### Experiment 2: impact of harmonization on a classification task

We applied the different harmonization methods to a tumor grading task (LGG vs. GBM) and evaluated their respective performance using several ML algorithms. These algorithms were implemented to verify that performance was not related to the type of algorithm used. The different algorithms that were selected were C-Support vector classification (SVC), k-nearest neighbors vote (KNN), logistic regression (LR), random forest (RF) and eXtreme Gradient Boosting (XGBoost). These classifiers are among the most used for supervised classification tasks and reflect the possible classification approaches with linear, non-linear, and ensemble classifiers. In the machine learning pipeline, min–max normalization of the radiomic features to the range 0–1 was included. Impact of the harmonization strategies was analyzed considering either the first-order features, second-order features or a combination of first-order features and second-order features as inputs of the ML models. Since the set of optimization spaces for the classifiers, ComBat and AutoComBat, was huge, a Bayesian optimization with a Gaussian process was implemented. During Bayesian optimization, 120 parameter settings were sampled and Balanced Accuracy was considered as the optimization metric. The parameter space used for Bayesian optimization is given in Table S2. We reported results for five different metrics: Area Under the Receiver Operating Characteristic Curve (ROC AUC), Balanced Accuracy, F1 score, Precision, and Recall. Please note that this experiment was independent from the previous one, meaning that batch assignment and alignment were done considering performance for the tumor grading task as the optimization metrics.

The scikit-learn^48^ v0.23.2 library and the scikit-optimize^63^ v0.8.1 library were used for the ML and Bayesian optimization pipelines respectively.

**Figure 2:**
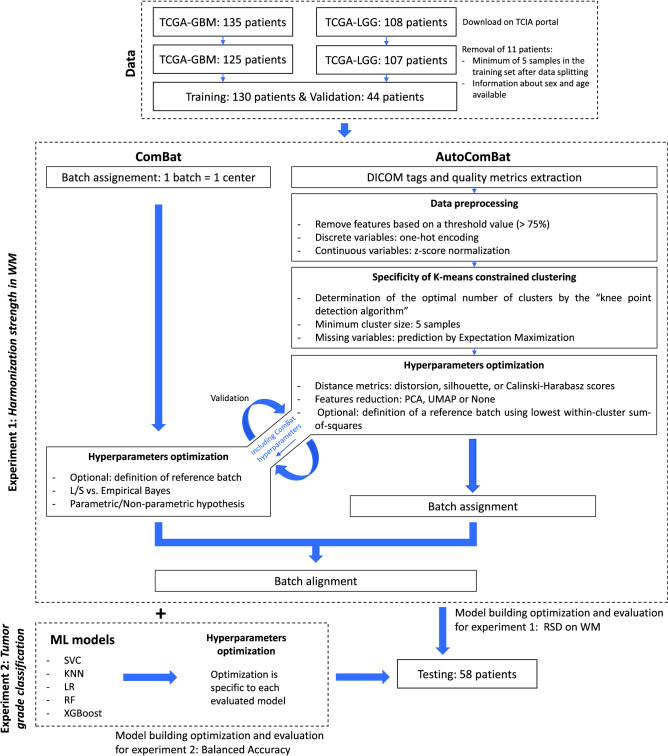
Workflows for the ComBat and AutoComBat computation models. These workflows are performed after extraction of the radiomic features.

The overall workflow for the ComBat and AutoComBat implementation is illustrated in Fig. 2.

## Results

### Illustration of the “batch effect”

**Figure 3:**
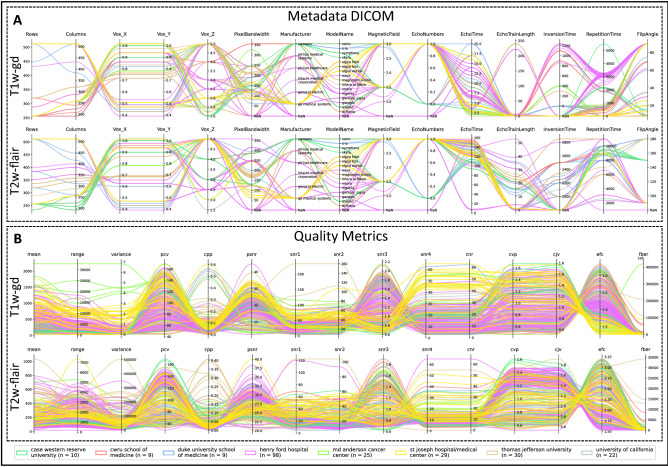
Parallel coordinate plots per center of the information extracted from the dataset for the T1w-gd and T2w-flair MRI. (**a**) Information extracted from the header of the DICOM files. (**b**) Measurement of quality metrics.

Figure 3 summarizes information extracted from the DICOM metadata and quality metrics derived from the raw DICOM images for T1w-gd and T2w-flair MR images. A similar plot is available for T1w and T2w MRI in Fig. S1. This parallel coordinate plot facilitates the visualization of multivariate data and the observation of trends. Figure 3A illustrates the difficulty of the task of assigning a batch label considering all acquisition parameters from the DICOM header. It highlights the fact that a given center may use multiple devices, such as for the T1w-gd with Henry Ford Hospital, which uses 8 different devices: GE Signa Excite (n = 31), GE Signa Genesis (n = 15), Philips Ingenia (n = 6), GE Signa HDxt (n = 5), Philips Intera (n = 1), Hitachi Oasis (n = 1), GE Signa HDx (n = 1), and NaN (n = 38), i.e., for which the information is not available and the St. Joseph Hospital which used 3 different devices: GE Signa Excite (n = 26), GE Signa HDxt (n = 2) and GE Signa HDx (n = 1). For a same device such as the GE Signa Excite and considering the T1w-gd images, the acquisition parameters may vary, e.g., the repetition time for the Henry Ford Hospital was 2989 ± 484 ms, while this value was 45 ± 122 ms for the St. Joseph Hospital. In Fig. 3B, image quality metrics were extracted in the considered population. For the GE Signa Excite device and T1w-gd MRI in the St. Joseph Hospital, SNR2 was equal to 46 ± 17 and EFC metric to 1.66 ± 0.42. Again, for Henry Ford Hospital, the corresponding values were respectively equal to 35 ± 9 and 2.12 ± 0.17.

### Evaluation of harmonization strength based on WM

**Figure 4:**
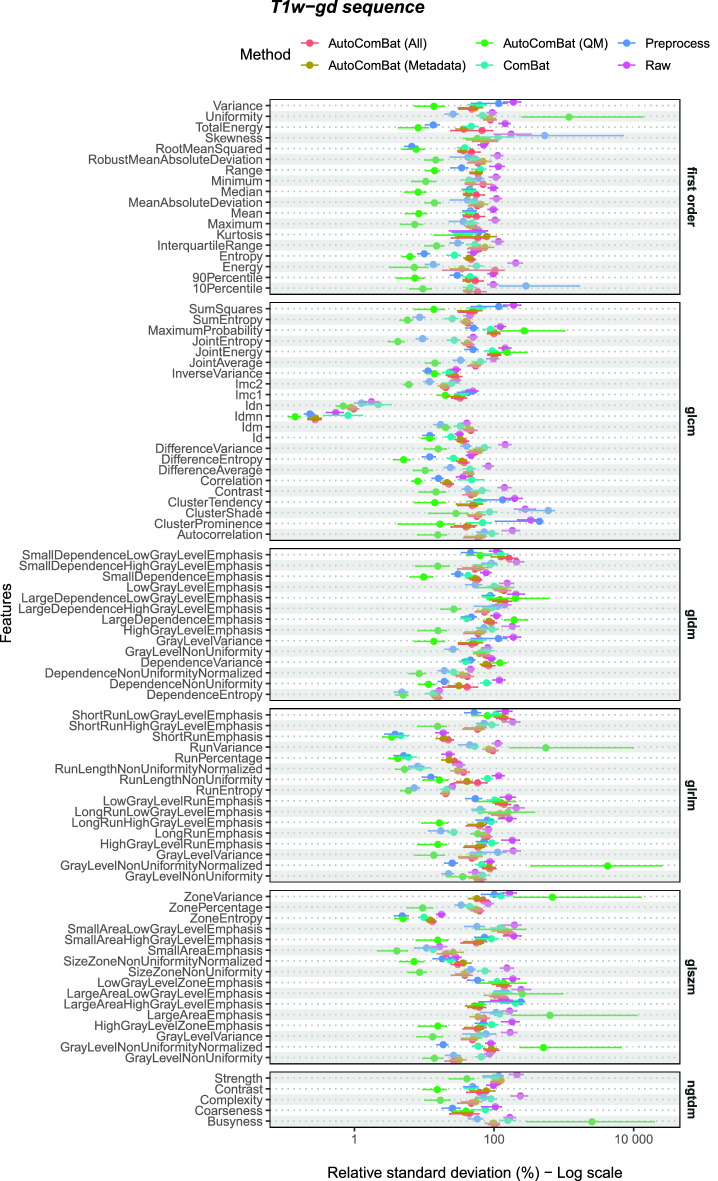
Harmonization strength evaluated on the WM radiomic features for the T1w-gd MRI on the test set. Points represent the RSD values and error bars are the 95% CI.

**Figure 5:**
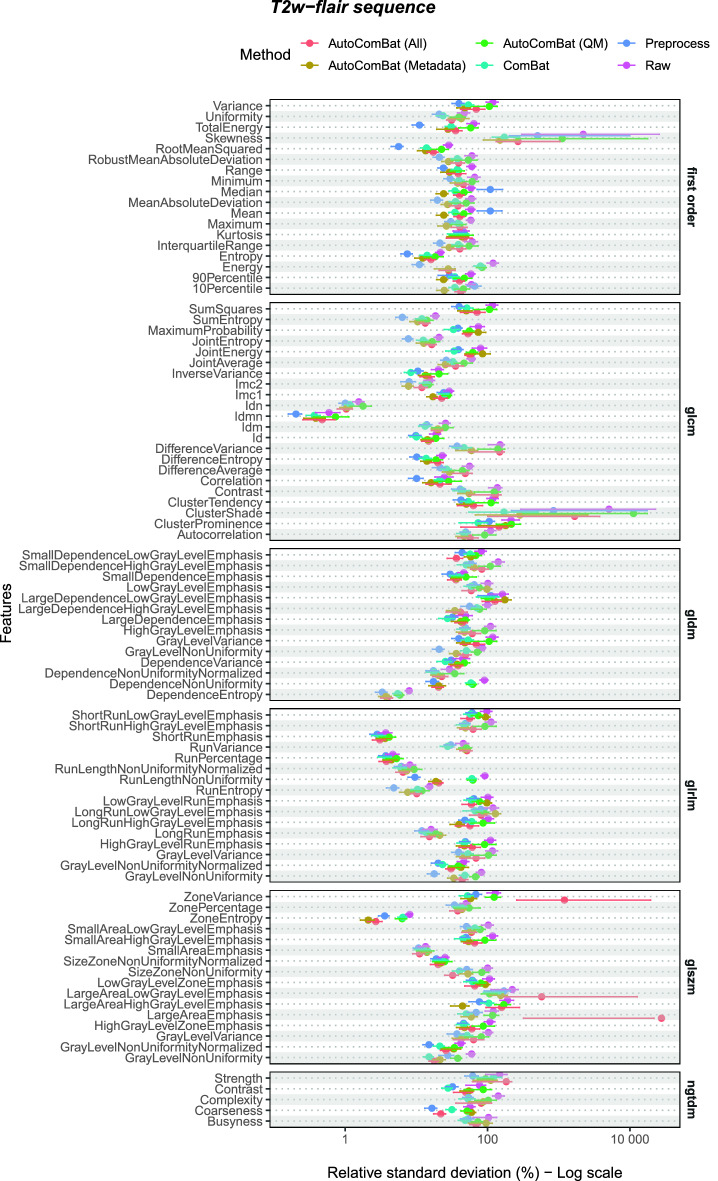
Harmonization strength evaluated on the WM radiomic features for the T2w-flair MRI on the test set. Points represent the RSD values and error bars are the 95% CI.

Table 3 summarizes the number of features leading to a RSD lower than the one obtained considering the raw MR images by feature class and MR image type (T1w-gd and T2w-flair) on the test set when applying optimal ComBat and AutoComBat methods. Data corresponding to the test set for T1w and T2w MRI and validation sets for all MRI types are also available in Tables S3 and S4, respectively. Figs. 4 and 5 illustrate the impact of the different harmonization strategies on the features extracted from the WM for the test set for T1w-gd and T2w-flair MRI respectively, Fig. S2 for the test for T1w and T2w MRI, and Fig. S3 for the validation sets for all MRI weightings. A method was considered as the best method (Top) if its RSD was the lowest, not included in the 95% CI of the raw data, and its 95% CI was not included in that of other methods. When multiple methods had overlapping CIs, they were all counted. In some cases, none of the methods met all three criteria, and in some cases more than one did, which explains the potential mismatch between the sum of the figures and the total number shown at the top of the column. In addition, the total number of significant features per method compared to the raw method is given. Comparing WM RSD of all methods to raw RSD, the preprocessing method showed the highest harmonization capabilities for T1w-gd (82%), while it was ComBat for T2w-flair (79%), very similar to preprocess (78%). For T1w and T2w, ComBat was found to be superior (96%—T1w; 92%—T2w), followed closely by the preprocessing method (93%—T1w; 85%—T2w). For AutoComBat, these values varied according to the characteristics used for the clustering (metadata and quality metrics, metadata only, quality metrics only) and the MR image type, but never outperformed ComBat, except for T1w-gd. For example, using all available, metadata only, and quality metrics only features for clustering, the number of significant features showed an improvement of 54%, 62%, 75% compared to raw, respectively. Looking at top features, AutoCombat using QM only obtained the best results for T1w-gd with 75% of features obtaining the lowest RSD. The preprocess method showed the best performance for T2w-flair with 78% of features. For MRI sequences not shown here (T1w and T2w in Supplementary), ComBat was superior, with AutoComBat QM presenting very similar values. These findings on the test set agreed with the validation set, except for AutoComBat (all) in the T2w-flair images, which had shown the best performance in the validation set but did not generalize the same way in the test set.

**Figure 6:**
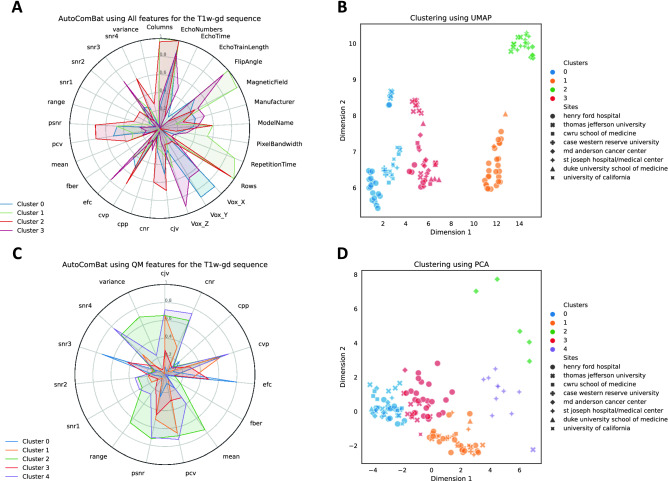
Clustering interpretation of AutoComBat for the T1w-gd images. (**A**, **C**) correspond to normalized feature importance in the final clusterings and (**B**, **D**) are visual representations of the proposed clusters. In both cases, a feature reduction strategy was retained in AutoComBat. (**A**, **B**) AutoComBat using all features as inputs - UMAP feature reduction, (**C**, **D**) AutoComBat using QM - PCA feature reduction.

Figure 6 attempts to interpret the clusters by showing the normalized feature importance of each feature when running AutoComBat on T1w-gd images. We remind that the variables have been previously scaled between 0 and 1. Fig. 6A,B consider all features (Metadata and QM), while Fig. 6C,D are focused on QM only. The selected hyperparameters were similar for both with $empirical\_bayes=True$, $parametric=True$, $use\_ref\_batch=True$, $metric=distortion$, except for the feature reduction method with UMAP for one (Fig. 6B) and PCA for the other (Fig. 6D). Based on Fig. 6A, we can see that 4 clusters were selected for RSD minimization considering all features. Cluster 2 contained only images with both a high number of rows and columns and low repetition time, which corresponded to data coming mainly from St Joseph hospital/medical center and Thomas Jefferson University (Fig. 6B), in accordance with the parallel coordinate plot (Fig. 3A). Similarly, in Fig. 6D, cluster 4 included mainly St Joseph hospital/medical center data corresponding to high SNR4, CNR, CVP, PCV, CJV with low EFC and variance whose trend can be followed in Fig. 3B.

**Table 3:** Counts (%) of features for each harmonization method with a RSD (95% CI) lower than the one corresponding to the raw images for the T1w-gd and T2w-flair MRI on the test set.

MRI	Method	Feature class						(n = 91)	
		First-order(n=18)	glcm(n=22)	gldm(n=14)	glrlm(n=16)	glszm (n=16)	ngtdm(n=5)		
								Total	
								Top	Vs. raw
*T1w-gd*	Preprocess	4	5	7	12	7	2	37 (41%)	75 (82%)
	ComBat	3	1	3	6	2	0	15 (16%)	62 (68%)
	AutoComBat								
	All	2	2	0	1	5	1	11 (12%)	49 (54%)
	Metadata	3	3	1	2	5	1	15 (16%)	56 (62%)
	QM	16	20	8	10	10	4	68 (75%)	68 (75%)
*T2w-flair*	Preprocess	13	20	11	10	13	4	71 (78%)	71 (78%)
	ComBat	10	14	11	9	11	4	59 (65%)	72 (79%)
	AutoComBat								
	All	6	5	8	7	8	2	36 (40%)	47 (52%)
	Metadata	12	12	6	4	10	1	45 (49%)	55 (60%)
	QM	2	0	2	0	1	0	5 (5%)	7 (8%)

The main part of the table gives the number of features for which the considered method is evaluated as the best one, which is called “Top”. Total vs. Raw gives the total number of features for each method that are significantly better compared to Raw.

For AutoComBat, “All” means the use of Metadata and Quality Metrics. *QM* Quality Metrics.

### Tumor grading performance

**Figure 7:**
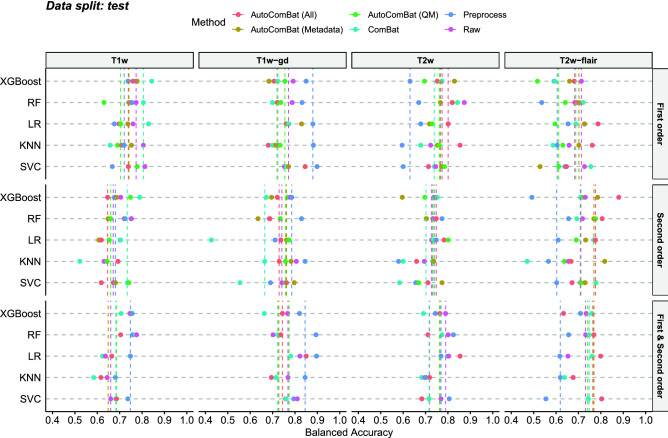
Balanced accuracy for the tumor grading task for the 5 machine learning models (RF, SVC, XGBoost, KNN, LR) and the different MRI images (T1w, T1w-gd, T2w, T2w-flair) on the test set for the first, second and first & second-order feature types depending on the harmonization method. Each color corresponds to a harmonization method. Each dot indicates the performance of one ML algorithm, and the vertical dashed line is the median value of the performance of the 5 ML algorithms.

Figure 7 summarizes the performance results of tumor grade classification in terms of balanced accuracy on the test set for the different MR images (T1w, T1w-gd, T2w, T2w-flair) considering either first-order, second-order feature classes only or a combination of both. For the T1w MR images and first-order features, ComBat ranked first with a median performance for the 5 algorithms of 0.81 (min: 0.66, max: 0.84), while all other methods ranged from 0.70-0.74. Raw data yielded a performance of 0.77 (min: 0.76, max: 0.81). For second-order features, ComBat also ranked first with a value of 0.73 (min: 0.52, max: 0.79), while all other methods ranged from 0.64-0.68. Raw data gave a performance of 0.67 (min: 0.62, max: 0.75). For the T1w-gd images and first-order features, image preprocessing gave the best performance with a value of 0.87 (min: 0.83, max: 0.90). The other methods ranged between 0.72-0.75 and use of raw images yielded a median balanced accuracy of 0.77 (min: 0.73, max: 0.79). For second-order features, preprocessing obtained the best performance with a value of 0.79 (min: 0.69, max: 0.85). Direct use of raw data and AutoComBat led to performance between 0.72 and 0.77, while ComBat underperformed with a value of 0.66 (min: 0.43, max: 0.74). For the T2w MR images and first-order features, AutoComBat (all), i.e., using all features available for clustering, ranked first with a value of 0.80 (min: 0.71, max: 0.85). Here, preprocessing performed worse with a value of 0.63 (min: 0.60, max: 0.68), while the others ranged between 0.74-0.77. Considering the second-order features, AutoCombat (all) took first position again with a value of 0.75 (min: 0.66, max: 0.78). Combat performed the worst with a value of 0.70 (min: 0.58, max: 0.75), while the rest including raw data ranged between 0.72-0.74. Finally, the development of a ML model based on T2w flair-extracted first-order features with AutoComBat (Metadata) and without applying any processing yielded values of 0.70 (min: 0.53, max: 0.73) and 0.71 (min: 0.66, max: 0.73), respectively. Here, AutoComBat (QM) performed worse with a median balanced accuracy of 0.61 (min: 0.52, max: 0.64). The value was equal to 0.61 (min: 0.64, max: 0.65) when preprocessing was applied. For the second-order features, AutoComBat (all) and AutoComBat (metadata) provided the best results with 0.78 (min: 0.67, max: 0.88) and 0.77 (min: 0.73, max: 0.82), respectively. Preprocessing obtained the worst results here with a value equal to 0.60 (min: 0.49, max: 0.66).

In supplementary, these results are also available for validation (Fig. S4). Results for additional metrics (F1 score, precision, recall and ROC auc) are also available in supplementary (Figs. S5–S12).

When considering all radiomic features as inputs of the ML models, preprocessing drastically outperformed the other harmonization methods when dealing with T1 weightings, whether or not a contrast agent is injected with median balanced accuracies respectively equal to 0.75 and 0.85 for T1w and T1w-gd images respectively. For T2w-flair MRI, the results are reversed in favor of ComBat. Interestingly, for this MR weighting, AutoCombat whether considering metadata plus quality metrics as inputs or metadata alone outperformed the traditional Combat, with median balanced accuracies equal to 0.77, 0.76 and 0.75 respectively.

## Discussion

The aim of this study was to analyze the impact of the harmonization approach in an MR-based radiomics context, i.e., either upstream as image processing or downstream after the extraction of radiomic features. In addition, a clustering method that aims to automatically define the batch to which an image should be assigned, using information from the DICOM file metadata and/or quality metrics deduced from the raw images themselves, was proposed. In this work, a highly heterogeneous dataset including conventional MR images (T1w, T1w-gd, T2w, T2w-flair) from a large number of centers was voluntary considered to evaluate the generalizability of the proposed solution, and both classes of radiomic features (first and second-orders) were analyzed separately first and in combination in a second step. Two types of experiments were conducted to quantify the impact of the harmonization strategy. In a first time, it was analysed on its ability to decrease RSD of radiomic features extracted from patches of the white matter over the whole patient cohort. Second, a clinical grading task (HGG vs. LGG) was considered.

Batch assignment is not a trivial task when data are very heterogeneous as illustrated in Figure 3, as no consensus international guidelines exist regarding acquisition parameters in brain oncology. Indeed, spatial resolution, signal to noise ratio and contrast to noise ratio strongly depend on field gradients, B0 magnetic field, pulse sequence and its parameters in MR^64,65^. Clustering based on DICOM file metadata and/or metrics was proposed here with the goal to minimize an objective function corresponding to the average of an RSD corresponding to 91 radiomic features extracted from WM. Image metrics have been introduced in addition to conventionally used DICOM tags to facilitate batch assignment in case of lack of information in the DICOM header or when the number of patients considered for a certain type of acquisition is too low. AutoComBat reveals coherent batch allocations as illustrated in Figure 6, without a total scattering of the centers in the different clusters, highlighting that whatever the manufacturer of the imaging device and its model, the centers have habits in the parameterization of their sequences. Considering the four MR images (results only showed for T1w-gd), image size (rows and columns), voxel size, magnetic field and flip angle parameters were shown to have the highest weights in the clustering. Although weights were dependent on the MR images considered, the metrics that most often appeared with significant weights were CJV, PCV, EFC, SNR and variance.

Applied to a tumor grading task, our methodology showed different results depending on the MR images and classes of features considered (first-order, second-order or both). For the T1w-gd images, often considered as the most informative in neuro-oncology, image preprocessing yields the best results with a median balanced accuracy equal to 0.87 considering the first order features only while others methods range between 0.72-0.75. For second-order features, preprocessing also gives the best results with a median balanced accuracy equal to 0.87. This result is the best over all combinations of MR images and harmonization methods and is generalizable, i.e., with no discrepancies between the validation and the test. This result was confirmed when dealing with combined first-order and second-order radiomic features as inputs of the ML models, allowing us to conclude that, for the considered task, preprocessing of MR images is the optimal way to standardize radiomic analyses when dealing with T1 weightings, whether or not a contrast agent is injected. However, preprocessing underperforms the other strategies for T2-weightings, which by nature suffer from limited intensity ranges. AutoComBat (based either on Metadata or all features) has shown interesting properties, especially on the T2w-flair image with the best median value of balanced accuracy equal to 0.78, considering second order radiomic features only as inputs. For this image weighting and feature class, AutoComBat has demonstrated a good generalization compared to other methods, i.e., constant performance between the validation and the test sets (only 5% percentage difference). Conventional Combat was the best method for the T1w images with a median balanced accuracy of 0.81 and 0.73 for the first and second-order features, respectively. This proof-of-concept therefore highlights the potential of AutoCombat for data harmonization, especially as it is applicable in very highly multi-constructed and multi-parametric contexts.

In the literature, only a few works have been dedicated to the use of ComBat in MRI and more specifically applied to radiomics. The first work, which tested the ComBat approach for a radiomic application in the case of MRI, used a rescan on two separate machines, with the unique difference being the magnetic field (1.5T vs. 3T)^34^. They evaluated the method on T2w-flair and T1w-gd MR images of 18 brain tumor patients with a limited set of 42 extracted radiomic features. The difference in harmonization realignment was quantified based on a Friedman test in two different regions (WM and tumor volume). Three types of images were considered to this: a raw image, an image normalized by the hybrid white stripe (hWS) method and resampled to a voxel size of 1x1x1 $mm^{3}$, and an image incorporating the previous steps but with the addition of ComBat. Using image preprocessing, an improvement of 19 percentage points for feature distribution realignment was found in WM regions and 38 percentage points in the tumor volume for the T2w-flair images compared to the raw images. By adding ComBat, they showed an improvement of 88 percentage points in WM regions of interest and 96 percentage points in tumor volume for the T2w-flair images. They concluded that image processing with the addition of ComBat completely eliminated the statistical differences between the radiomic features extracted from images acquired at 1.5T and 3T. Compared to Orlhac et al.^34^ study, Combat was applied on raw images directly in the present work, with almost identical results: we have shown that 68% and 79% of the features for the T1w-gd and T2w-flair images, respectively, yielded a harmonization strength augmentation in the WM. We think that the strength of ComBat is that it should learn some sources of variabilities, thus bypassing some preprocessing steps. Nevertheless, it remains interesting to include image resampling before features extraction so that the texture features remain rotation invariant and the correction of artifacts as bias field correction.

The interest in the ComBat approach was also evaluated in the recent study of Da-Ano et al.^35^, where four versions of the nonparametric ComBat were compared in their ability to harmonize radiomic features in a multicenter context, including two clinical datasets. The first dataset was composed of 119 patients suffering from locally advanced cervical cancer and contained MR and PET images from three different centers. The second involved 98 patients with locally advanced laryngeal cancer from 5 centers who underwent contrast-enhanced computed tomography. Among the four versions, one version identified a reference center, in addition to the conventional version, on which radiomic features were transformed. The other two versions used conventional versions, but with the addition of Boostrap and Monte-Carlo strategies for improved robustness in the estimation. They showed that all four versions of ComBat showed a contribution in removing machine differences, and improving the prediction performance of the given outcome. In addition, the version using a reference site gave the best results. For example, Modified ComBat resulted in a 6% improvement in balanced accuracy compared to untransformed data for the random forest algorithm in the prediction of local failure in locally advanced cervical cancer. When using ComBat in the 5 centers dataset, they were confronted with the fact that the machine parameters were very heterogeneous. Following this observation, they would have had to manually assign a batch to each image, leading to more than 15 labels, which they did not consider realistic due to the limited number of patients. We have shown from Figs. 3 and S1 that there is limited sense to affect to a same batch images coming from a single center but for which devices or acquisition parameters differ, even though centers tend to harmonize meaningful parameters in terms of image interpretation as shown earlier. This study, therefore, highlighted the urgent need to define an alternative for batch assignment, as already mentioned. For this purpose, Da-Ano et al.^35^ proposed an unsupervised hierarchical clustering technique applied directly to radiomic features. Using this technique, they were able to correctly cluster the patients in the dataset from the three centers with homogeneous acquisition parameters per center into three different clusters. Only one patient was misclassified. Then, they applied clustering to the dataset with heterogeneous parameters to establish the ComBat “batch” labels. We believe that the direct use of radiomic features extracted from the tumor itself to define a “batch” could be biased by the clinical endpoint and lead to clusters correlated to the outcome. In their case, however, they tested the hypothesis by verifying that each resulting group had a similar percentage of non-responders. Using either information extracted from the DICOM headers and characterizing machine and parameters variability and/or using image metrics seems to be a better way to categorize images without any assumption. Another study used the ComBat approach with the goal to develop a model capable of capturing the relationship between image quality metrics and relative volume corrections for each region of the brain^66^. They demonstrated that the tool could reduce systemic scanner variations in new images from unknown scanners. This work supports the notion that identifying the “batch” with data that are irrelevant to the problem we are trying to solve and therefore unrelated to the clinical outcome of interest is promising.

In addition, to propose a generalizable alternative for batch allocation, the present study also gives tracks about the correct use of ComBat in a machine learning process applied to radiomics. We would like indeed to warn the community about the misuse of ComBat in several radiomics studies. This error, which consists in pooling all the data (train, val, test) and applying ComBat, leads to data leakage. In fact, as with any application of a normalization step in machine learning, it is indeed important to normalize data after their splitting to avoid introducing future information into the training explanatory variables (i.e., the mean and variance). Our code available at the following address https://github.com/Alxaline/ComScan answers this problem by following the philosophy of scikit-learn with a fit and transform function. The hardest part in using Combat is that there are not always ground truths about the batch labels, in particular in the case of very heterogeneous data as it is in a multicentric context. The advantage of using clustering to determine the batch is that it becomes possible to know whether the imaging data not seen during the training stage lies outside the distribution of the training data. This does not solve the generalizability problem in a general way but gives an idea of the space in which the imager must be located for a developed radiomic signature to be applicable.

This study poses some limitations. First, the clustering method was limited to a constrained K-means, but other methods could be considered, such as Density-Based Spatial Clustering of Applications with Noise (DBSCAN). However, the proposed method has the advantage of not requiring to specify a priori the number of clusters, takes as argument the minimal sample, i.e., the smallest number of points needed to form a cluster, and is robust to noise. For AutoComBat, all the potential was not exploited because we were limited by the comparison with ComBat, which necessitates balancing patients between the sets depending on their origin center. For the same reason, we were limited to a simple data splitting strategy and were not able to use cross-validation, which would have limited overfitting. However, we have exploited the full potential of ComBat by exploring its complete hyperparameter space (reference site or not, parametric assumption or not, empirical Bayes strategy or not). As well, ComBat was applied in a very heterogeneous context, with stratification by institution, rather than by MR provider/software/sequence parameters, which is a highly unfavorable case, even if already used as such in recent studies^40^. We did not consider the discretization step as a variable parameter and have fixed it to a fixed bin width^29,67^. For the classification step, we did not try to establish the best model but put the emphasis on understanding the influence of each strategy on the radiomic features harmonization; that is why we have created separate models with either first-order or second-order features. Furthermore, we did not evaluate the shape features, which can also be affected by the acquisition parameters. Finally, the potential of AutoComBat should be further investigated for other datasets and other clinical tasks.

## Supplementary Information

Supplementary Information 1.
