# Supplementary material for: AutoComBat: a generic method for harmonizing MRI-based radiomic features
Source: Sci Rep. 2022 Jul 26;12:12762. doi: 10.1038/s41598-022-16609-1 (PMC9325761; doi:10.1038/s41598-022-16609-1)
Supplement: Supplementary file 1 — Supplementary Information 1. [file 41598_2022_16609_MOESM1_ESM.pdf]

Supplementary information

Table S1. Parameters space used for the grid search for the ComBat and AutoComBat methods

Method	Parameters		
	Name	*Space*	
**ComBat**	empirical_bayes	[True]	[False]
	parametric	[True, False]	
	ref_batch	[0, 1, 2, 3, 4, 5, 6, 7, None]	
**AutoComBat***	empirical_bayes	[True]	[False]
	parametric	[True, False]	
	use_ref_batch	[True, False]	
	metric	[distortion, silhouette, calinski_harabasz]	
	features_reduction	[PCA, UMAP, None]	

* For AutoComBat, the number of components were fixed to 2 when a features reduction was applied.

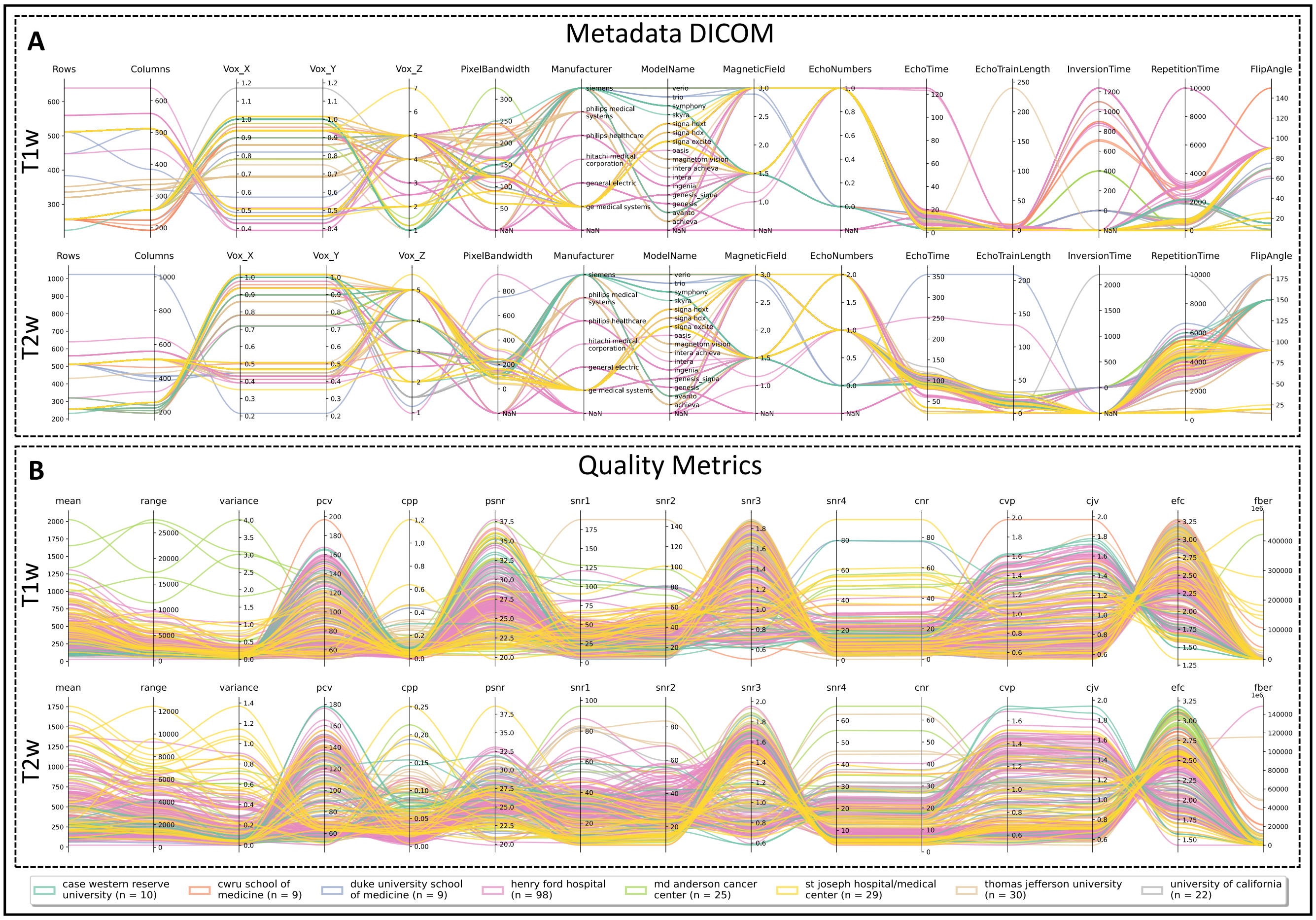

Figure S1. Parallel coordinate plots per center of the information extracted from the dataset for the T1w and T2w MRI sequences. a) Information extracted from the header of the DICOM files. b) Measurement of Quality Metrics.

Table S2. Parameters space used for the Bayesian optimization of the different ML models

ML models	Parameters		
	Name	***Space***	***Distribution***
**SVC**	C	[1e-6, 1e+4]	log-uniform
	gamma	[1e-6, 1e+1]	log-uniform
	degree	[1, 8]	uniform
	kernel	[linear, poly, rbf]	-
**KNN**	n_neighbors	[1, 20]	uniform
	weights	[uniform, distance]	-
	algorithm	[auto, ball_tree, kd_tree, brute]	-
	metric	[euclidean, manhattan, minkowski]	-
	leaf_size	[1, 50]	uniform
	p	[1, 4]	uniform
**LR**	C	[1e-5, 100]	log-uniform
	solver	[newton-cg, lbfgs, liblinear]	-
	penalty	[None, l1, l2, elasticnet]	-
	fit_intercept	[True, False]	-
**RF**	n_estimators	[100, 2500]	uniform
	class_weight	[None, balanced]	-
	max_features	[auto, sqrt, log2]	-
	max_depth	[10, 150]	uniform
	min_samples_split	[1, 10]	uniform
	min_samples_leaf	[1, 10]	uniform
	criterion	[gini, entropy]	-
	bootstrap	[True, False]	-
**XGBoost**	learning_rate	[0.01, 1.0]	log-uniform
	min_child_weight	[0, 10]	uniform
	max_depth	[0, 50]	uniform
	max_delta_step	[0, 20]	uniform
	subsample	[0.01, 1.0]	uniform
	colsample_bytree	[0.01, 1.0]	uniform
	colsample_bylevel	[0.01, 1.0]	uniform
	reg_lambda	[1e-9, 1000]	log-uniform
	reg_alpha	[1e-9, 1.0]	log-uniform
	gamma	[1e-9, 0.5]	log-uniform
	n_estimators	[50, 100]	uniform
	scale_post_weight	[1e-6, 500]	log-uniform

Notes.
ComBat and AutoComBat corresponds to the space defined in Table S1. An exception appears for AutoCombat, where the space of the number of components was in the range of 2 to 5 when a feature reduction technique was used.

Table S3. Counts (%) of features for each harmonization method with a RSD (95% CI) lower than the one corresponding to the raw images for the T1w and T2w sequences on the test set. The main part of the table gives the number of features for which the considered method is evaluated as the best one, which is called "Top". Total vs. Raw gives the total number of features for each method that are significantly better compared to Raw.

MRI	Method		first order (n=18)	glcm (n=22)	gldm (n=14)	glrlm (n=16)	glszm (n=16)	ngtdm (n=5)	Total (n=91) Top	Total (n=91) vs. Raw
T1w										
	Preprocess		9	10	**11**	**13**	9	**3**	55 (60%)	85 (93%)
	ComBat		11	**15**	10	**13**	**12**	2	**63 (69%)**	**87 (96%)**
	AutoComBat	All	8	4	3	3	3	0	21 (23%)	39 (43%)
		Metadata	1	4	0	1	2	0	8 (9%)	26 (29%)
		QM	**16**	**15**	8	8	10	**3**	60 (66%)	61 (67%)
T2w										
	Preprocess		14	9	8	8	8	**3**	50 (55%)	77 (85%)
	ComBat		**15**	12	**11**	**12**	**13**	3	**66 (73%)**	**84 (92%)**
	AutoComBat	All	15	5	4	4	4	0	32 (35%)	52 (57%)
		Metadata	**15**	4	4	5	4	1	33 (36%)	48 (53%)
		QM	15	**13**	7	10	7	**3**	55 (60%)	59 (65%)

Notes.
For AutoComBat, "All" means the use of Metadata and Quality Metrics. QM = Quality Metrics.

Table S4. Counts (%) of features for each harmonization method with a RSD (95% CI) lower than the one corresponding to the raw images for each MRI sequences on the validation set. The main part of the table gives the number of features for which the considered method is evaluated as the best one, which is called "Top". Total vs. Raw gives the total number of features for each method that are significantly better compared to Raw.

	Method		Feature class							
			first order (n=18)	glcm (n=22)	gldm (n=14)	glrlm (n=16)	glszm (n=16)	ngtdm (n=5)	(n=91)	
**MRI**									**Total**	
T1w									**Top**	**vs. Raw**
	Preprocess		13	14	9	14	9	**3**	62 (68%)	87 (96%)
	ComBat		**15**	**16**	**11**	**15**	**12**	2	**71 (78%)**	**89 (98%)**
	AutoComBat	All	4	5	2	5	3	1	20 (22%)	31 (34%)
		Metadata	3	6	3	5	3	0	20 (22%)	35 (38%)
		QM	**15**	16	9	12	**12**	**3**	67 (74%)	72 (79%)
T1w-gd										
	Preprocess		4	6	8	**12**	8	2	40 (44%)	**81 (89%)**
	ComBat		2	0	2	4	1	0	9 (10%)	37 (41%)
	AutoComBat	All	3	2	2	1	5	1	14 (15%)	51 (56%)
		Metadata	4	2	2	2	5	1	16 (18%)	54 (59%)
		QM	**16**	**20**	**10**	11	**13**	**3**	**73 (80%)**	74 (81%)
T2w										
	Preprocess		10	**13**	4	5	6	2	40 (44%)	56 (62%)
	ComBat		**15**	**13**	**11**	**11**	**11**	3	**64 (70%)**	**73 (80%)**
	AutoComBat	All	13	11	4	4	3	1	36 (40%)	55 (60%)
		Metadata	14	7	5	4	5	1	36 (40%)	60 (66%)
		QM	**15**	**13**	**11**	**11**	**11**	**3**	55 (60%)	57 (63%)
T2w-flair										
	Preprocess		**13**	**17**	**9**	10	8	**4**	**61 (67%)**	62 (68%)
	ComBat		9	12	7	9	**10**	2	49 (54%)	56 (62%)
	AutoComBat	All	11	14	**9**	**11**	**10**	3	58 (64%)	**68 (75%)**
		Metadata	10	10	5	4	9	1	39 (43%)	50 (55%)
		QM	6	3	2	3	3	1	18 (20%)	21 (23%)

Notes.
For AutoComBat, "All" means the use of Metadata and Quality Metrics. QM = Quality Metrics.

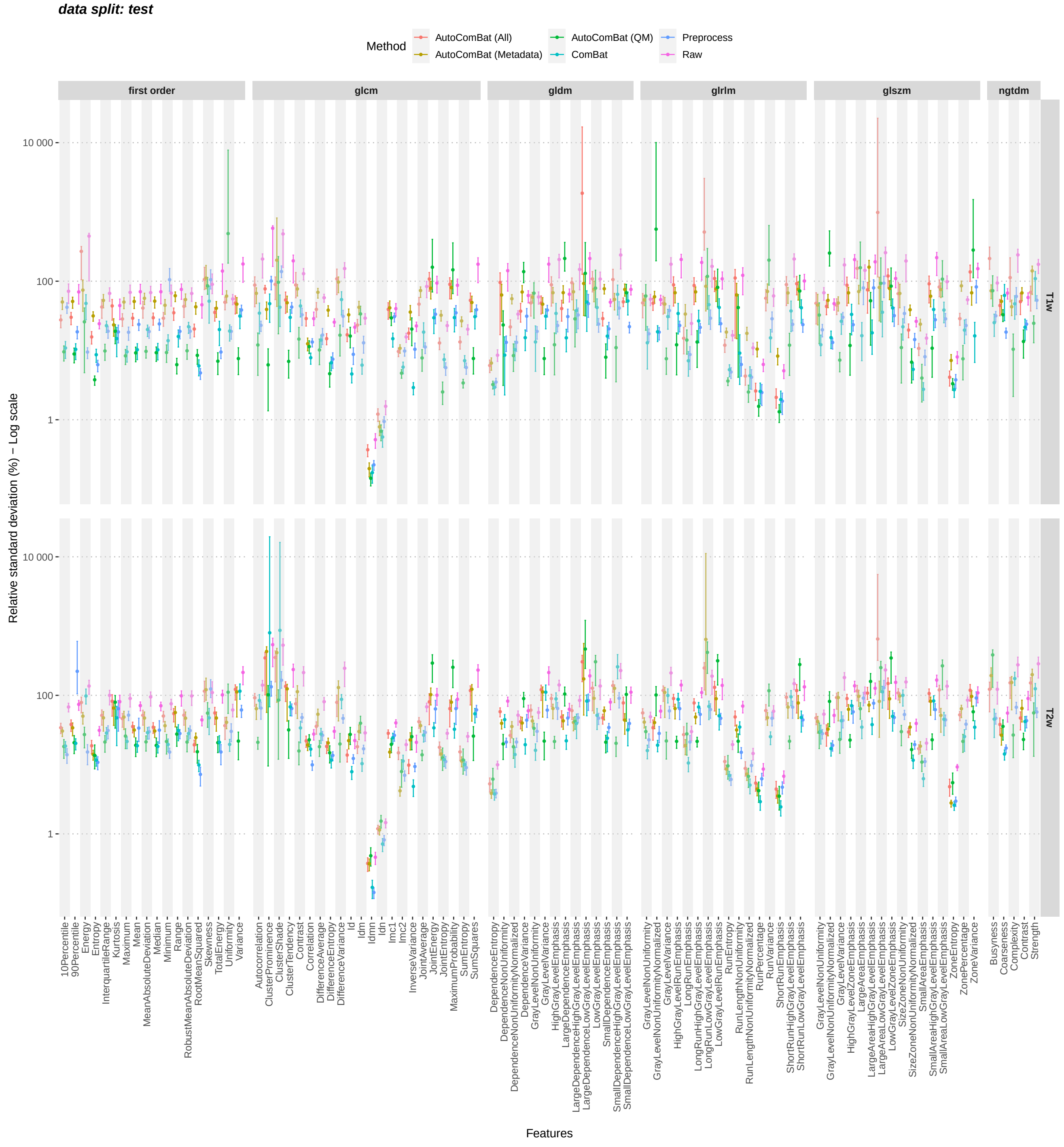

Figure S2. Harmonization strength evaluated on the WM features (column) for the different MRI sequences (T1w, first row, and; T2w, second row) on the test set. Point represents the RSD value and, error bar is the 95% CI.

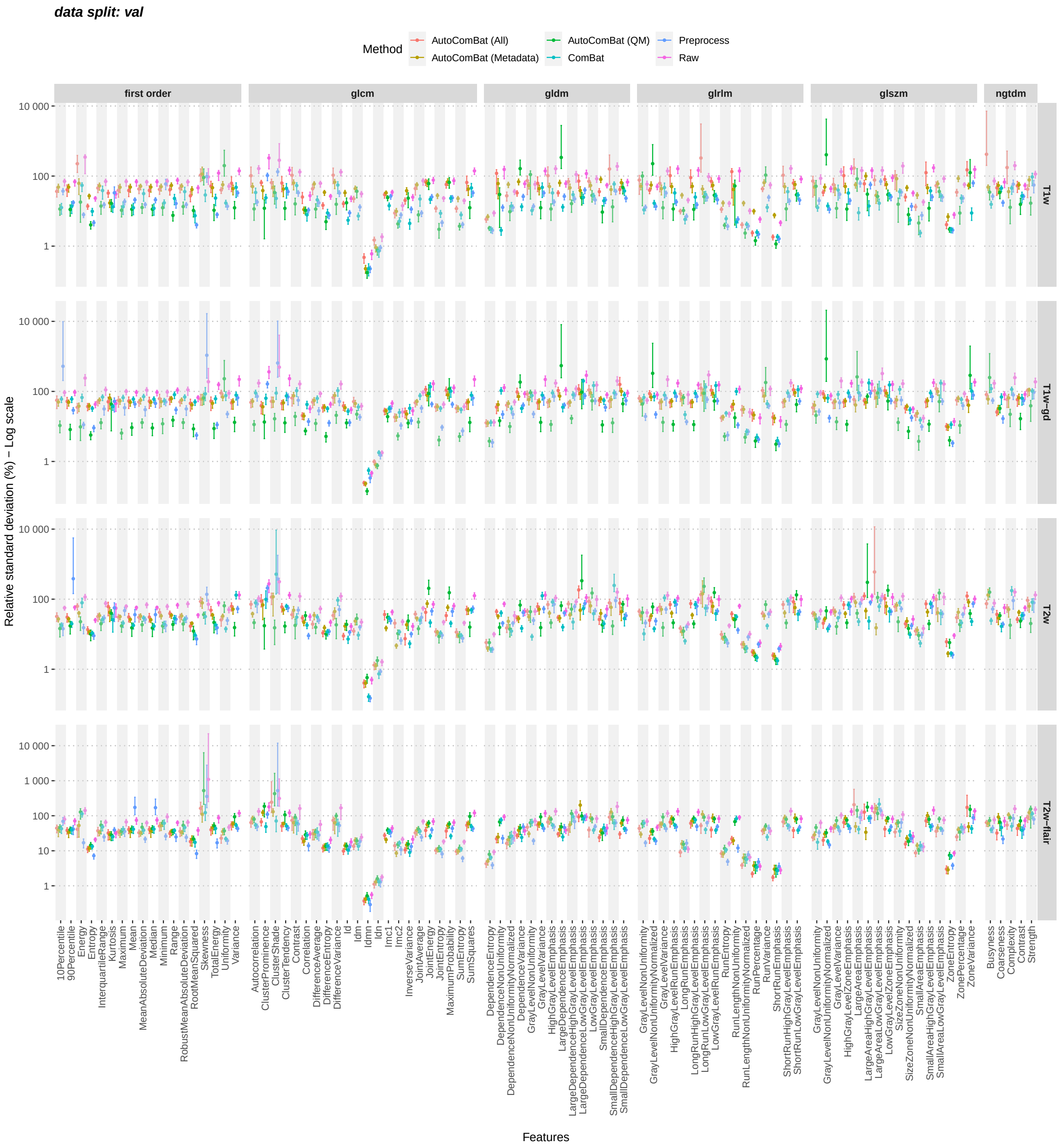

Figure S3. Harmonization strength evaluated on the WM features (column) for the different MRI sequences (T1w, first row; T1w-gd, second row; T2w, third row, and; T2w-flair, fourth row) on the validation set. Point represents the RSD value, and error bar is the 95% CI.

Data split: val

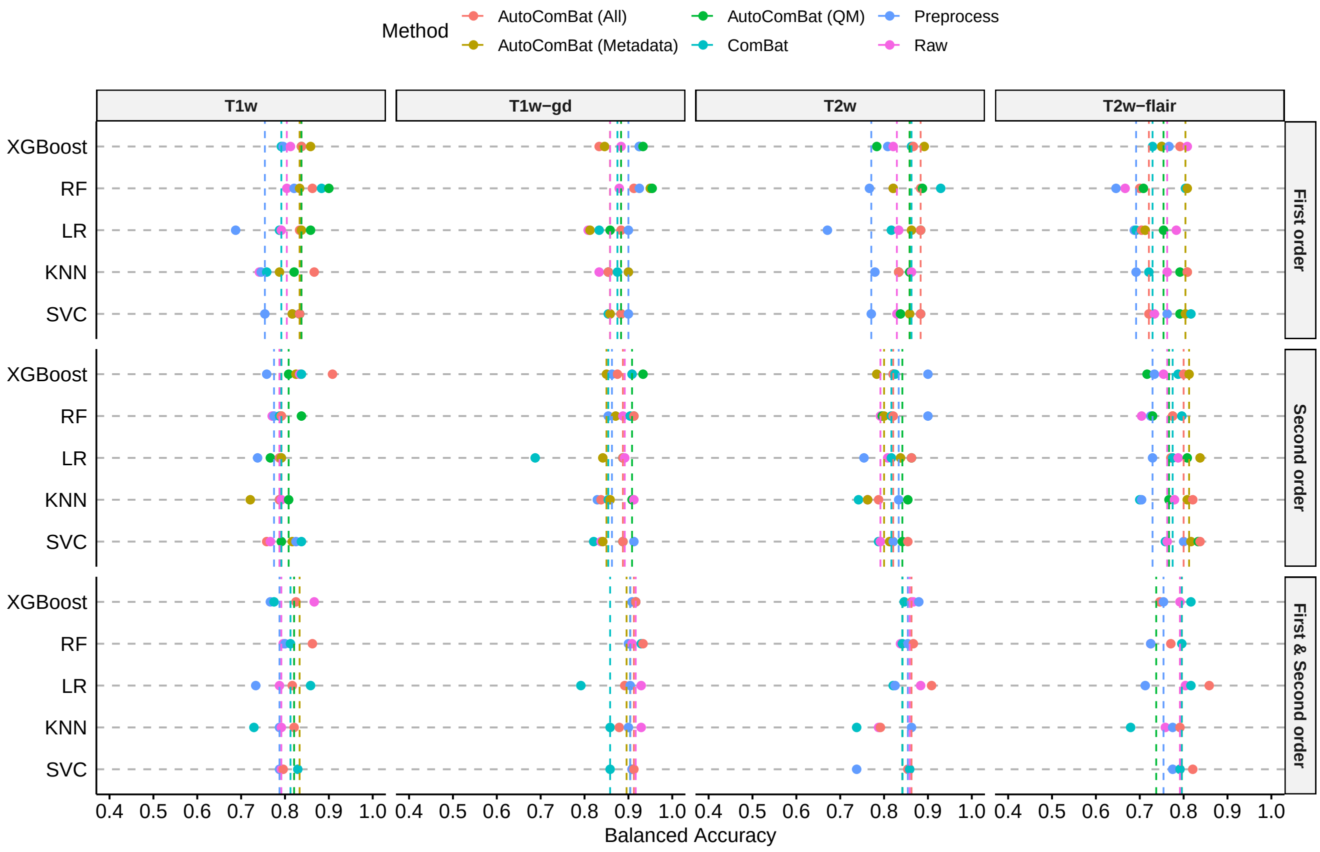

Figure S4. Balanced accuracy for the tumor grading task for the 5 machine learning models (RF, SVC, XGBoost, KNN, LR) and the different MRI sequences (T1w, T1w-gd, T2w, T2w-flair) on the validation set for the first, second and first & second-order feature types depending on the harmonization method. Each color corresponds to a harmonization method. Each dot indicates the performance of one ML algorithm, and the vertical dashed line is the median value of the performance of the 5 ML algorithms.

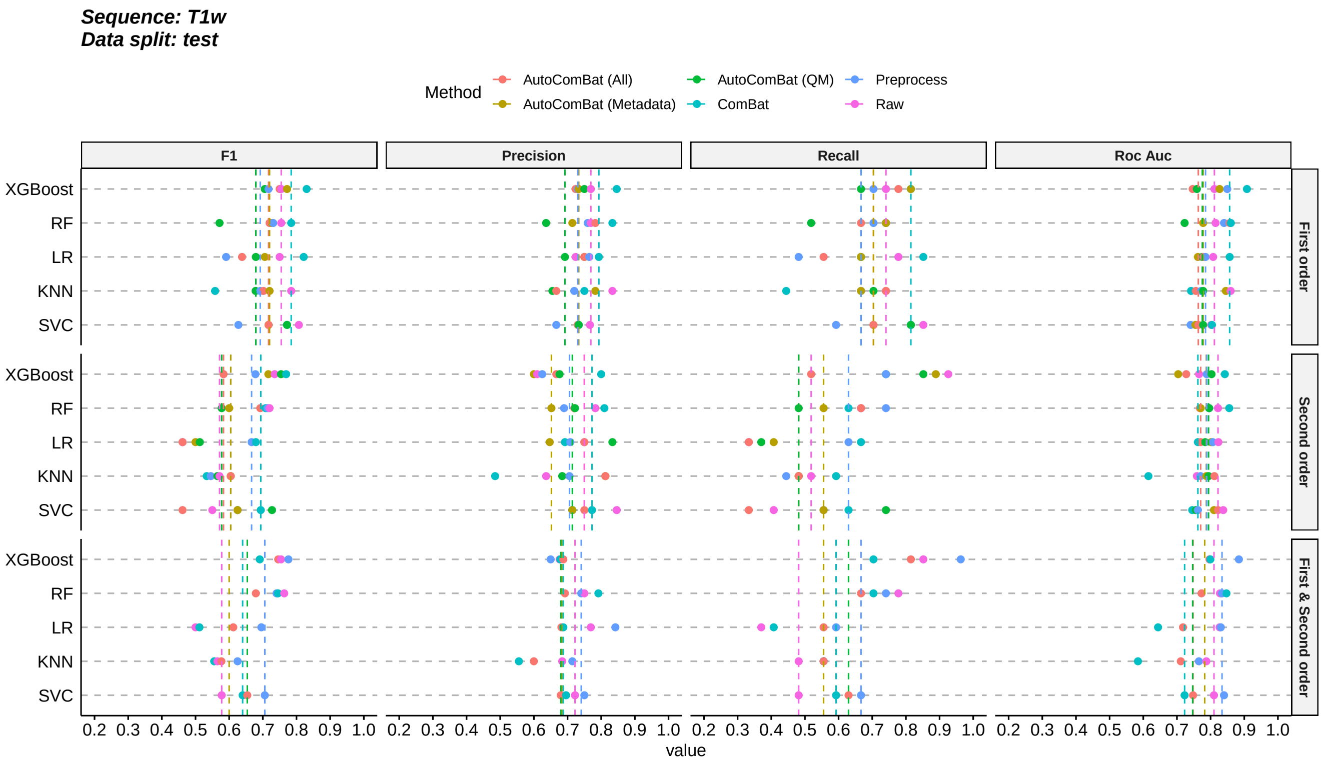

Figure S5. F1 score, Precision, Recall and Roc Auc for the tumor grading task for the 5 machine learning models (RF, SVC, XGBoost, KNN, LR) on the T1w MRI sequence on the test set for the first, second and first & second-order feature types depending on the harmonization method. Each color corresponds to a harmonization method. Each dot indicates the performance of one ML algorithm, and the vertical dashed line is the median value of the performance of the 5 ML algorithms.

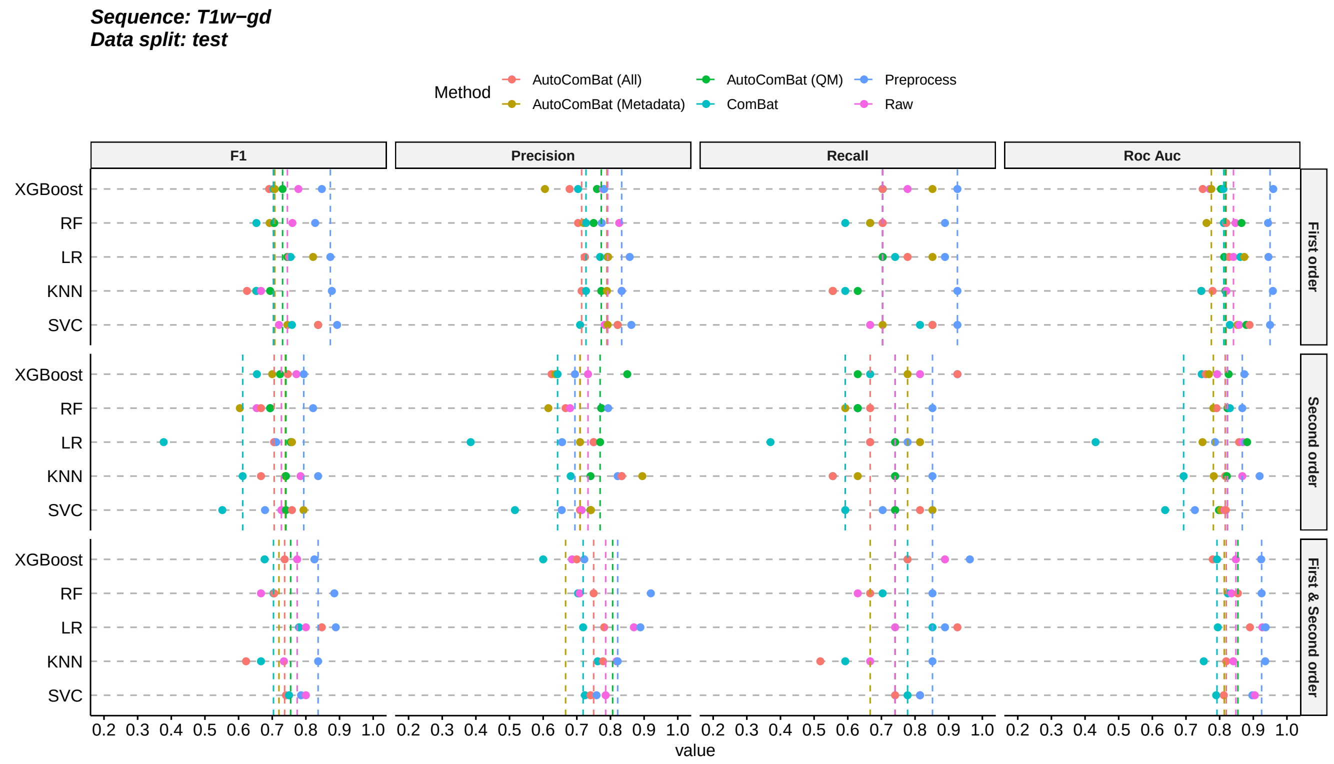

Figure S6. F1 score, Precision, Recall and Roc Auc for the tumor grading task for the 5 machine learning models (RF, SVC, XGBoost, KNN, LR) on the T1w-gd MRI sequence on the test set for the first, second and first & second-order feature types depending on the harmonization method. Each color corresponds to a harmonization method. Each dot indicates the performance of one ML algorithm, and the vertical dashed line is the median value of the performance of the 5 ML algorithms.

Sequence: T2w
Data split: test

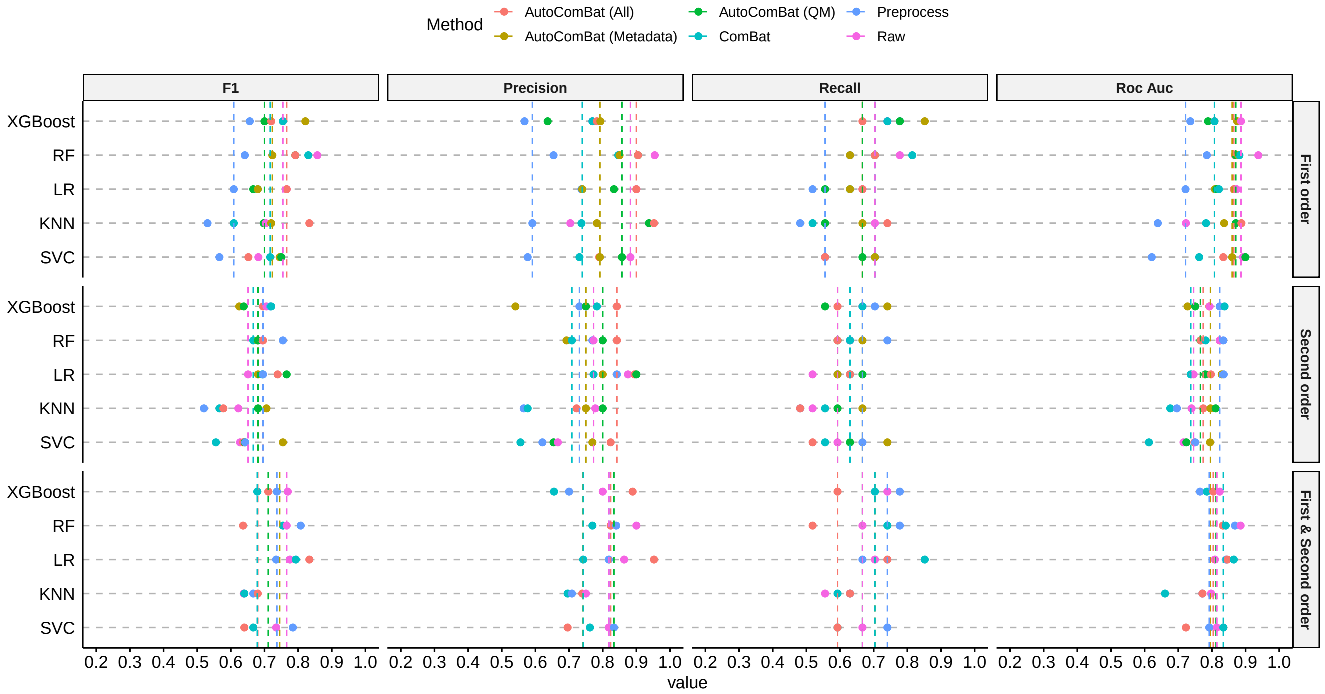

Figure S7. F1 score, Precision, Recall and Roc Auc for the tumor grading task for the 5 machine learning models (RF, SVC, XGBoost, KNN, LR) on the T2w MRI sequence on the test set for the first, second and first & second-order feature types depending on the harmonization method. Each color corresponds to a harmonization method. Each dot indicates the performance of one ML algorithm, and the vertical dashed line is the median value of the performance of the 5 ML algorithms.

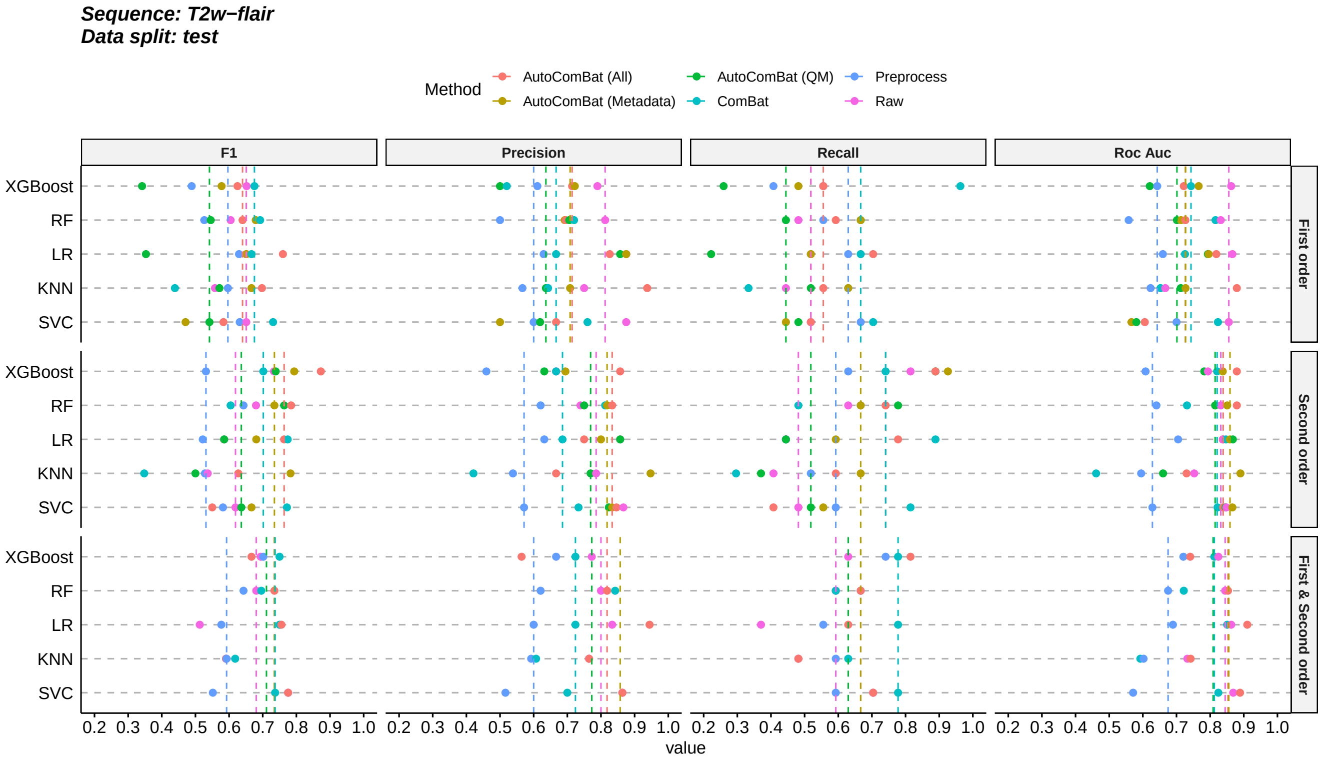

Figure S8. F1 score, Precision, Recall and Roc Auc for the tumor grading task for the 5 machine learning models (RF, SVC, XGBoost, KNN, LR) on the T2w-flair MRI sequence on the test set for the first, second and first & second-order feature types depending on the harmonization method. Each color corresponds to a harmonization method. Each dot indicates the performance of one ML algorithm, and the vertical dashed line is the median value of the performance of the 5 ML algorithms.

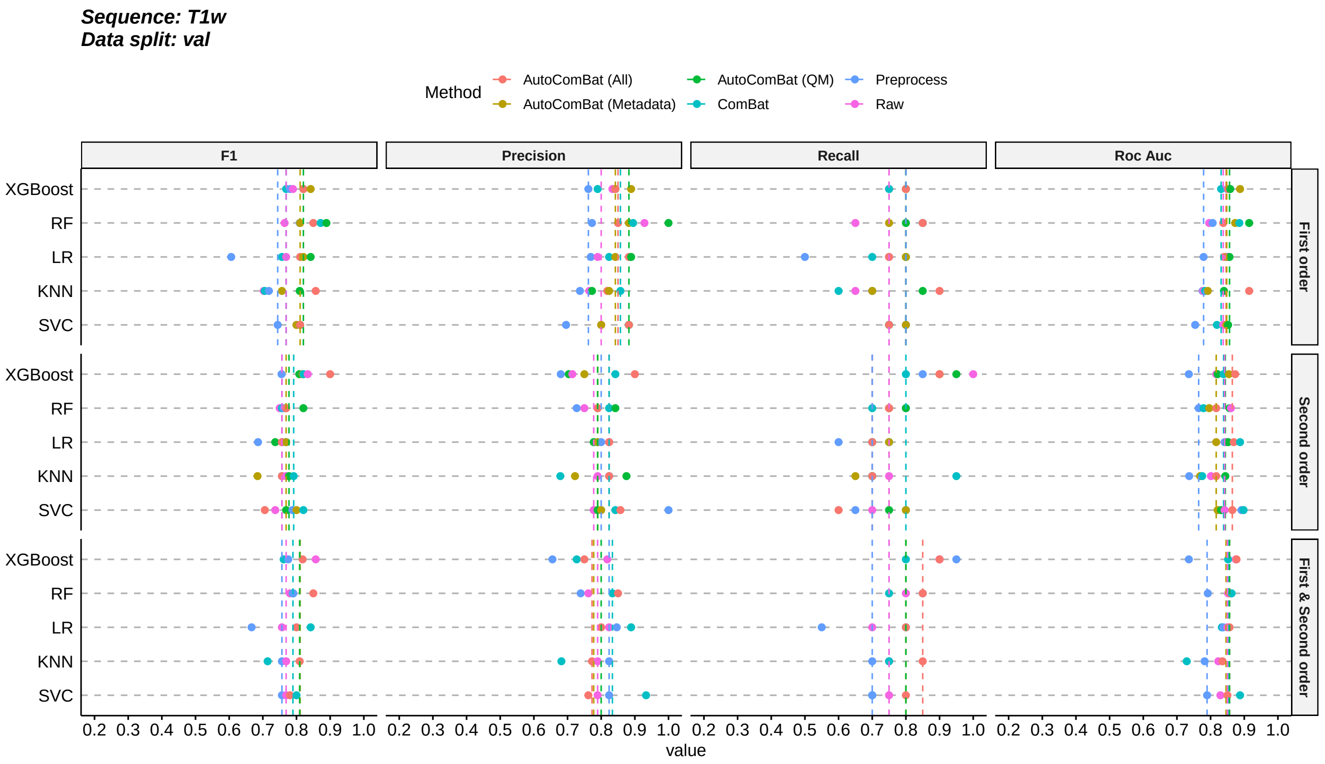

Figure S9. F1 score, Precision, Recall and Roc Auc for the tumor grading task for the 5 machine learning models (RF, SVC, XGBoost, KNN, LR) on the T1w MRI sequence on the validation set for the first, second and first & second-order feature types depending on the harmonization method. Each color corresponds to a harmonization method. Each dot indicates the performance of one ML algorithm, and the vertical dashed line is the median value of the performance of the 5 ML algorithms.

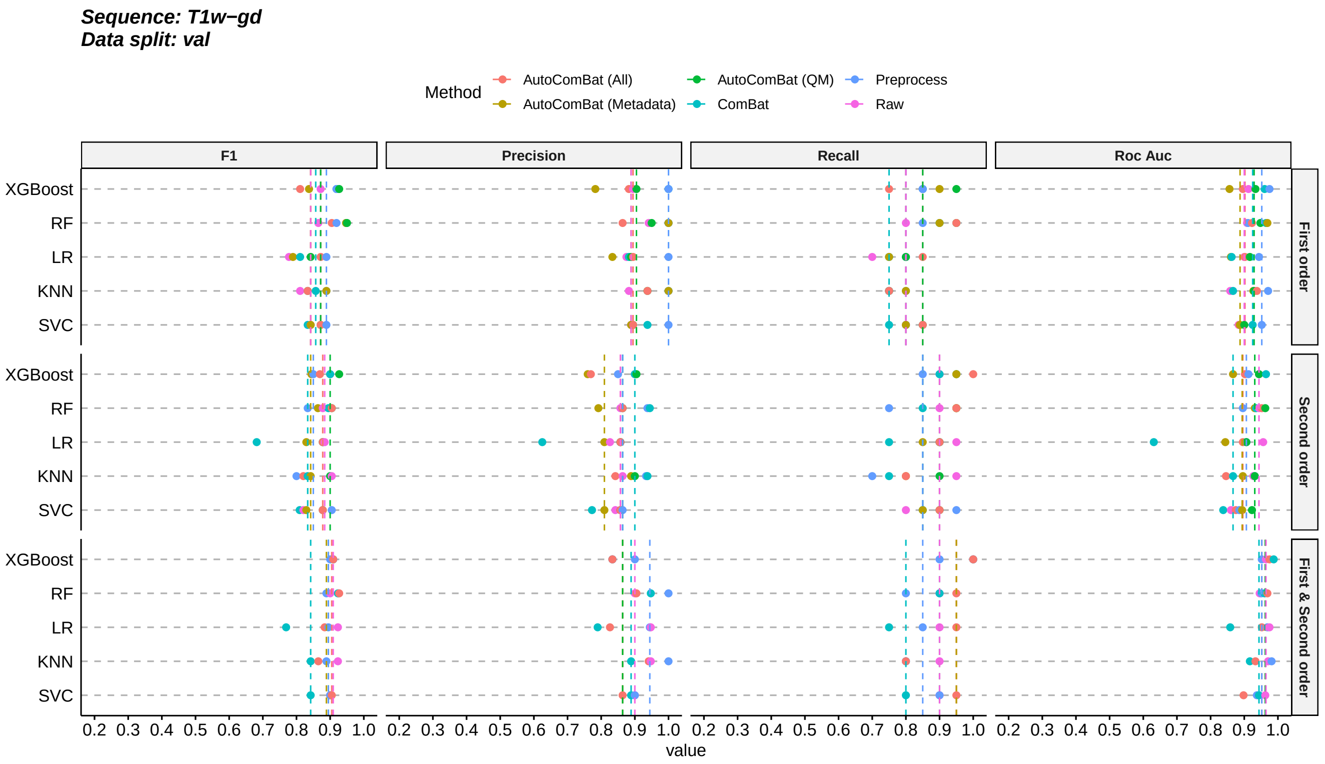

Figure S10. F1 score, Precision, Recall and Roc Auc for the tumor grading task for the 5 machine learning models (RF, SVC, XGBoost, KNN, LR) on the T1w-gd MRI sequence on the validation set for the first, second and first & second-order feature types depending on the harmonization method. Each color corresponds to a harmonization method. Each dot indicates the performance of one ML algorithm, and the vertical dashed line is the median value of the performance of the 5 ML algorithms.

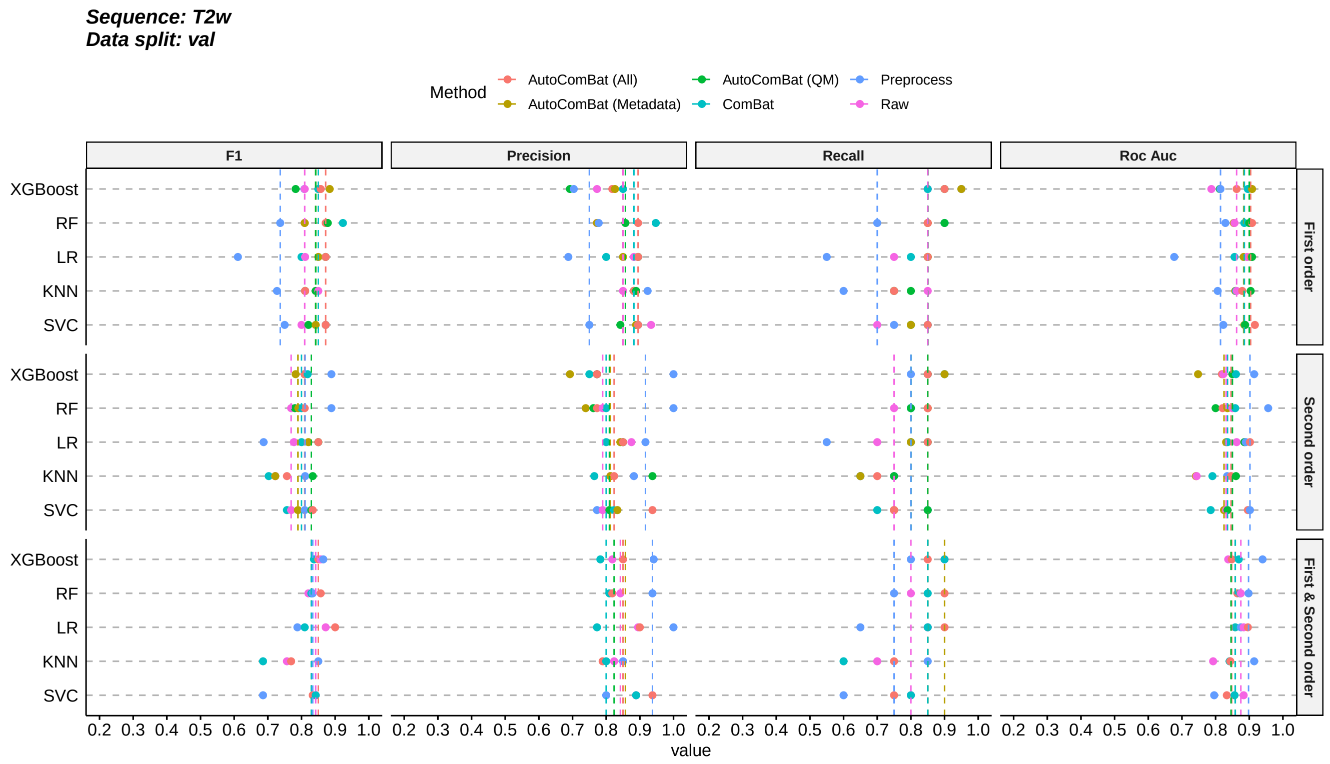

Figure S11. F1 score, Precision, Recall and Roc Auc for the tumor grading task for the 5 machine learning models (RF, SVC, XGBoost, KNN, LR) on the T2w MRI sequence on the validation set for the first, second and first & second-order feature types depending on the harmonization method. Each color corresponds to a harmonization method. Each dot indicates the performance of one ML algorithm, and the vertical dashed line is the median value of the performance of the 5 ML algorithms.

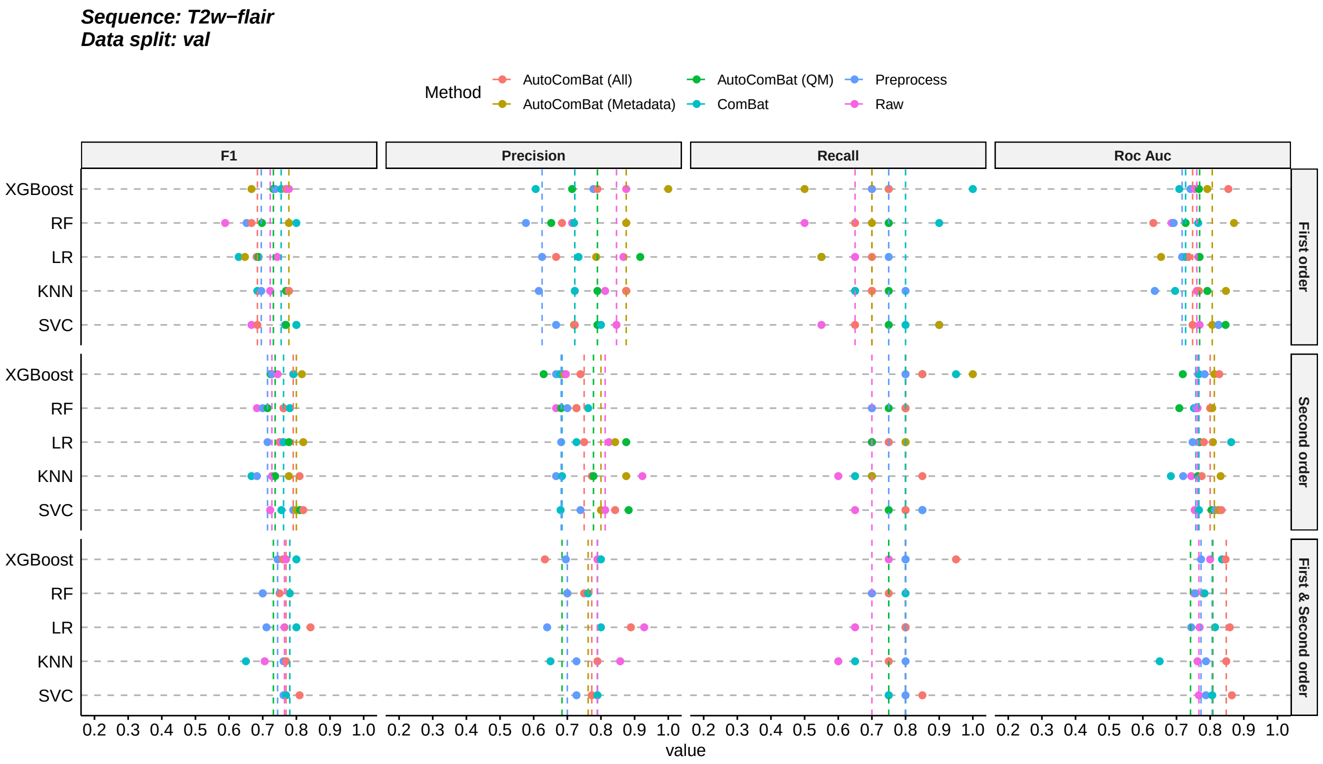

Figure S12. F1 score, Precision, Recall and Roc Auc for the tumor grading task for the 5 machine learning models (RF, SVC, XGBoost, KNN, LR) on the T2w-flair MRI sequence on the validation set for the first, second and first & second-order feature types depending on the harmonization method. Each color corresponds to a harmonization method. Each dot indicates the performance of one ML algorithm, and the vertical dashed line is the median value of the performance of the 5 ML algorithms.
